# Niacin and *B. animalis* subsp. *lactis* F1-7 Additively Attenuate Alcohol-Induced Liver Injury in a Gut Microbiota-Dependent Manner via the HCAR2-NLRP3 Pathway

**DOI:** 10.3390/nu18213487

**Published:** 2026-10-23

**Authors:** Xi Liang, Tianhu Shan, Jianhao Gao, Haichao Wen, Jiawen Wang, Zhe Zhang, Huaqi Zhang, Hui Liang

**Affiliations:** 1 College of Public Health, Qingdao University, Qingdao 266071, China; tianhu0427@163.com (T.S.); gaojh1009@163.com (J.G.); wenhc@qdu.edu.cn (H.W.); wangjiawen2018@163.com (J.W.); huaqi_erin@163.com (H.Z.); qdlianghui@126.com (H.L.); 2 College of Food Science and Engineering, Ocean University of China, Qingdao 266100, China; zhangzhe1613@ouc.edu.cn

**Keywords:** alcoholic liver injury, niacin, *Bifidobacterium animalis*, gut microbiota, β-hydroxybutyrate

## Abstract

Objectives: This study explored whether the combined administration of niacin and *B. animalis* subsp. *lactis* F1-7 exerts a protective effect against alcohol-induced liver injury via the gut-liver axis, with a specific focus on the HCAR2-NLRP3 signaling pathway, and further investigated the functional roles of gut microbiota and metabolites. Methods: A mouse model of alcoholic fatty liver disease was established via chronic ethanol feeding with the Lieber–DeCarli liquid diet. Experimental animals were divided into six intervention groups: control group, model group, niacin alone, F1-7 alone, combined niacin and F1-7 (NF), and antibiotic pretreatment followed by NF intervention. Results: The NF intervention significantly improved serum lipids, reduced ALT/AST, alleviated hepatic steatosis and inflammation, and restored intestinal barrier integrity, while upregulating HCAR2 and suppressing NLRP3 activation; these effects were largely abolished by antibiotics. The 16S rRNA sequencing revealed that NF intervention reshaped the gut microbiota, enriching beneficial genera such as *Lactobacillus*, *Bifidobacterium*, and *Faecalibaculum*. Untargeted metabolomics showed that NF intervention enriched the ketone body β-hydroxybutyrate, which was positively correlated with the abundance of beneficial bacteria; this metabolite was identified as a key node in the ketone-body synthesis/degradation pathway and ranked among the top differential metabolites by VIP score. Conclusions: The combination additively alleviates alcohol-induced hepatic and intestinal injury in a gut microbiota-dependent manner. Intestinal β-hydroxybutyrate was enriched following the combined intervention in a microbiota-dependent fashion, and this enrichment was associated with HCAR2 activation and NLRP3 inhibition. These findings demonstrate that the protective action is largely dependent on an intact gut microbiota and support the combined intervention as a promising strategy against alcoholic liver disease.

## 1. Introduction

Alcoholic liver disease (ALD), also known as alcohol-induced liver injury, refers to liver diseases caused by chronic and excessive alcohol consumption [1]. Epidemiologically, approximately 2.3 billion people consume alcoholic beverages, among which over 950 million engage in excessive drinking patterns [2]. During alcohol metabolism, the metabolites of alcohol induce inflammatory cytokine production and oxidative stress in hepatocytes, ultimately causing structural and functional hepatocyte damage [3].

Niacin (vitamin B3 or nicotinic acid), a water-soluble essential nutrient, acts as a potent ligand for the hydroxycarboxylic acid receptor 2 (HCAR2, previously designated GPR109A) [4]. Existing studies have demonstrated that niacin supplementation can ameliorate alcohol-induced intestinal barrier injury and hepatic inflammation, significantly reshape the gut microbiota, increase the abundance of colonic *Lactobacillus*, and promote the production of the metabolite butyrate [5]. HCAR2, as the receptor for niacin, is expressed on dendritic cells, macrophages, and intestinal epithelial cells, where it participates in the production of IL-8 and IL-10 and influences Treg cells, thereby alleviating or reducing inflammatory responses [6]. The activation of HCAR2 in the intestine is mainly regulated by gut microbial metabolites [7]. HCAR2 is an important receptor mediating the biological effects of niacin, butyrate, β-hydroxybutyrate and other substances [8]. In recent years, accumulating evidence has confirmed that ligand-bound HCAR2 exerts multiple biological functions, including lipid-lowering and anti-inflammatory effects, providing new therapeutic strategies for various diseases [9,10]. HCAR2 inhibits adenylyl cyclase via G_i/o proteins, reduces cAMP levels, and consequently suppresses NF-κB nuclear translocation, thereby decreasing the transcriptional priming of NLRP3 and pro-IL-1β [11]. Butyrate inhibits NLRP3 inflammasome activation via HCAR2 to ameliorate acute pancreatitis and associated intestinal injury in mice [12]. In animal models of alcohol-induced liver injury, β-hydroxybutyrate inhibits neutrophil infiltration and promotes the polarization of hepatic macrophages toward an anti-inflammatory M2 phenotype in an HCAR2-dependent manner [13].

The NLRP3 inflammasome is a cytoplasmic multiprotein complex in innate immunity composed of the sensor NLRP3, the adaptor ASC, and the effector caspase-1. Under homeostatic conditions, NLRP3 maintains an autoinhibited conformation through its PYD, NACHT, and LRR domains [14]. Upon sensing danger signals, NLRP3 oligomerizes and recruits ASC and pro-caspase-1, promoting autoproteolytic cleavage of pro-caspase-1 into active p10/p20 subunits; these subunits subsequently mediate IL-1β and IL-18 maturation and gasdermin D cleavage, triggering cytokine release and pyroptosis [15]. In alcohol-induced liver injury, ethanol is metabolized by alcohol dehydrogenase and CYP2E1 to acetaldehyde with substantial ROS production. This activates NLRP3 through multiple pathways, including direct activation by DAMPs, TLR4/NF-κB priming, and the gut–liver axis amplification loop in which alcohol disrupts the intestinal barrier, induces dysbiosis, and allows LPS to enter the liver via the portal vein to activate TLR4/NLRP3, thereby forming a vicious cycle of intestinal injury and hepatic inflammation. This leads to caspase-1-dependent pyroptosis and massive release of IL-1β/IL-18, making NLRP3 a key mediator in the progression of ALD from steatosis to alcoholic hepatitis and liver fibrosis [16].

Probiotics have been demonstrated to exert beneficial effects on the whole organism by improving digestion and immunity [17]. Bifidobacteria are naturally present in the colonic microbiota and contribute to nutrient absorption, intestinal homeostasis, immune enhancement, and anti-aging effects [18]. Upon colonizing the gut, *Bifidobacterium* promote the accumulation of metabolites such as indole-3-lactic acid. This compound inhibits the polarization of Th2 and Th17 effector cells, consequently boosting the synthesis of immunoregulatory cytokines like IL-27 and interferon-β, thereby sustaining immunological homeostasis within the gut environment [19]. The combined use of *Bifidobacterium* and *Lactobacillus* can modulate short-chain fatty acids via the gut-liver axis and activate HCAR2 to ameliorate hepatic lipid accumulation and inflammatory responses [20]. In our previous work, we identified a strain, *B. animalis* subsp. *lactis* F1-7, which plays an important role in preserving the microbial community structure in the terminal ileum and colon, as well as in exerting anti-inflammatory and lipid-lowering effects [21]. Our previous work has also established that niacin attenuates ethanol-induced hepatic injury and preserves gut microbial equilibrium via diverse mechanisms. For instance, niacin mitigates liver fibrosis by bolstering the antioxidant defense network, interfering with HSP90 chaperone function, promoting STAT3 ubiquitination and degradation, and attenuating signaling via the JAK1/STAT3 axis [22]. Meanwhile, after combined intervention with niacin and *B. animalis* subsp. *lactis* F1-7, we observed effective lipid-lowering effects [23]. However, the anti-inflammatory effects of this combination on the liver and intestine have not yet been elucidated.

In the current work, we developed a murine model of chronic alcohol feeding for 8 weeks to simulate alcohol-induced liver injury. The majority of published studies utilizing the Lieber–DeCarli ethanol feeding protocol, which is the standard model for early-stage alcoholic liver disease, employ male C57BL/6 mice aged 6–8 weeks to ensure model stability and consistency. The 8-week feeding duration in our study was specifically chosen to reproduce the early stages of chronic human ALD, including steatosis, oxidative stress, and liver injury [24]. Using niacin and B. animalis subsp. lactis F1-7 as interventions, we aimed to evaluate their combined anti-inflammatory effect against alcohol-induced hepatic damage. Although niacin and probiotics have each shown promise in ALD, it remains unknown whether their combination produces additive protection through a microbiota-dependent mechanism, and which specific microbial metabolite mediates this effect. We further sought to determine whether shifts in the intestinal metabolome following the dual intervention act on HCAR2 to confer protection against ALD via the gut-liver axis. In particular, it has not been established whether the combination of niacin and F1-7 acts additively through a microbiota-dependent metabolite–HCAR2 pathway or whether an intact gut microbiota is causally required for this protection. Dietary modulation of the gut ecosystem is a promising strategy for metabolic and inflammatory disorders and provides a framework for understanding microbiota-dependent nutritional interventions such as niacin plus probiotics [25,26]. To address this gap, we generated pseudo-germ-free animals through administration of a broad-spectrum antibiotic mixture and incorporated an experimental arm receiving both niacin and *B. animalis* subsp. *lactis* F1-7. Deciphering this mechanistic pathway is of paramount importance for understanding the contribution of microbiota-derived molecules to host metabolic control and will furnish a conceptual foundation for creating functional combination products aimed at alleviating alcohol-related liver insult.

## 2. Materials and Methods

### 2.1. Strain Culture

*B. animalis* subsp. *lactis* F1-7 (CCTCC M 2020830) was preserved at the China Center for Type Culture Collection. The microorganism was initially propagated in MRS broth (Qingdao Haibo Biotechnology Co., Ltd., Qingdao, China) at 37 °C. Following two rounds of subculturing for activation, a 1% (*v*/*v*) inoculum was introduced into fresh MRS liquid medium and cultivated at 37 °C over a 48-h period. The resulting biomass was harvested via centrifugation at 4 °C and 1500× *g* for 15 min. The bacterial precipitate was rinsed twice with phosphate-buffered saline (PBS). The bacterial suspension was adjusted to a final density of 1 × 10^9^ CFU/mL based on plate counts on MRS agar. The gavage volume was 0.2 mL per mouse per day, corresponding to a delivered dose of 2 × 10^8^ CFU per mouse per day.

### 2.2. Animal Grouping and Experimental Design

The number of animals per group was prospectively determined in accordance with the 3Rs principle of Reduction and the ARRIVE 2.0 guidelines. An a priori power analysis (G*Power 3.1.9.7; one-way ANOVA, six groups; effect size f = 0.40 derived from our previous studies using the same Lieber–DeCarli model and interventions; α = 0.05; power = 0.80) yielded a minimum of approximately 10 mice per group after Tukey adjustment. Accounting for an approximately 20% attrition rate during the 8-week feeding period (ethanol-related mortality and gavage-related loss, based on pilot experiments), 15 mice per group were assigned. The 90 SPF male C57BL/6 mice, aged 8 weeks and weighing 20 ± 2 g, were procured from Beijing Vital River Laboratory Animal Technology Co., Ltd. (Approval No. SCXK (Beijing, China) 2016-0006). The animals were accommodated under regulated environmental conditions with consistent temperature and humidity, subjected to a 12-h light/12-h dark cycle, and housed within the Animal Experiment Center of Qingdao University. All procedures involving animals adhered to the regulations stipulated by the Institutional Animal Ethics Committee and received authorization from the Experimental Animal Welfare and Ethics Committee of Qingdao University (Authorization No. QDU-AEC-2025308). Mice were randomly divided into six groups (*n* = 15 per group): control group (Control), alcoholic fatty liver disease model group (AFLD), niacin group (NA), *B. animalis* subsp. *lactis* F1-7 group (F1-7), niacin + *B. animalis* subsp. *lactis* F1-7 group (NF), and antibiotic + niacin + *B. animalis* subsp. *lactis* F1-7 group (NF-ABX). The Control group was fed an alcohol-free Lieber–DeCarli liquid diet. The NA, F1-7, NF, and NF-ABX groups were fed a Lieber–DeCarli liquid diet containing 4% ethanol (40 g ethanol per liter of liquid diet). Control and AFLD groups: 10 mL/kg saline by gavage; NA group: 50 mg/kg niacin by gavage; F1-7 group: 1 × 10^9^ CFU/mL by gavage (0.2 mL/day); NF group: 50 mg/kg niacin + 1 × 10^9^ CFU/mL F1-7 by gavage. The NF-ABX group first received an antibiotic cocktail (0.2 g/L ampicillin, 0.2 g/L neomycin, 0.2 g/L metronidazole, and 0.1 g/L vancomycin) in drinking water for 2 weeks to deplete the gut microbiota. After this 2-week antibiotic pretreatment, antibiotic administration was discontinued, and the mice then entered the 8-week experimental period together with all other groups, receiving the same combined intervention as the NF group (50 mg/kg niacin plus 1 × 10^9^ CFU/mL *B. animalis* subsp. *lactis* F1-7 by gavage) while being fed the 4% ethanol Lieber–DeCarli diet. Gavage was performed once daily.

### 2.3. Sample Collection and Processing

At the conclusion of the 8-week experimental period and following the final gavage administration, freshly voided fecal pellets were gathered into cryogenic vials and immediately frozen in liquid nitrogen. All mice underwent a 12-h fasting period with unrestricted access to water. After recording body weight, anesthesia was induced via intraperitoneal injection of sodium pentobarbital (40 mg/kg). Euthanasia was performed by intraperitoneal overdose of sodium pentobarbital followed by exsanguination via retro-orbital bleeding, and death was confirmed by cervical dislocation before tissue collection. Whole blood was collected via retro-orbital bleeding and centrifuged at 4 °C and 3500× *g* for 15 min to isolate serum for subsequent biochemical analyses. Liver specimens were excised, weighed, and a portion was preserved in fixative for histological assessment. Segments of the ileum, colon, and their luminal contents were also harvested. All biological specimens were maintained at −80 °C.

### 2.4. Serum Lipid Detection

Quantification of serum total cholesterol (TC), triglycerides (TG), high-density lipoprotein cholesterol (HDL-C), and low-density lipoprotein cholesterol (LDL-C) was performed using commercially available biochemical assay kits (Nanjing Jiancheng Bioengineering Institute, Nanjing, China).

### 2.5. Liver Hematoxylin–Eosin (HE) Staining and Oil Red O Staining

Hepatic pathological alterations were evaluated via hematoxylin–eosin (H&E) and Oil Red O staining. Briefly, for H&E assessment, liver specimens were fixed in 4% paraformaldehyde, processed through graded ethanol dehydration and xylene clearing, and subsequently embedded in paraffin. Deparaffinized sections (5 μm) were stained sequentially with hematoxylin and eosin, then mounted with neutral resin. For visualization of neutral lipids via Oil Red O, fresh liver tissue was cryo-embedded in OCT compound and sectioned at 10 μm. Following removal of the embedding medium, sections were incubated with Oil Red O solution at ambient temperature, counterstained with Mayer’s hematoxylin, and coverslipped using glycerol gelatin.

### 2.6. Serum Inflammatory Cytokine Detection

Circulating concentrations of LPS, TNF-α, IL-1β, and IL-18 were determined using commercial ELISA kits (Shanghai Jianglai Biotechnology Co., Ltd., Shanghai, China). The detection ranges were 12.5–800 pg/mL for LPS, 31.25–2000 pg/mL for TNF-α, 12.5–800 pg/mL for IL-1β, and 7.81–500 pg/mL for IL-18. The intra-assay coefficients of variation (CVs) were all <8%, and the inter-assay CVs were all <12%, as reported by the manufacturer. Assays were performed according to the manufacturer’s protocols. Briefly, standards and samples were added to pre-coated plates along with detection antibody, followed by incubation at 25 °C with agitation. After sequential washing, streptavidin-HRP conjugate and chromogenic substrate were applied. The enzymatic reaction was terminated with stop solution, and optical densities were recorded at 450 nm and 630 nm using a microplate reader. Sample concentrations were extrapolated from standard curves.

### 2.7. Liver Inflammation Detection

Liver tissue homogenates were prepared and centrifuged to obtain supernatants. ALT and AST levels were measured according to the manufacturer’s instructions for the kits Nanjing Jiancheng Bioengineering Institute, Nanjing, China) to assess the degree of liver inflammatory injury.

### 2.8. Immunohistochemical Detection of Intestinal Mucosal Barrier Proteins

Alterations in intestinal mucosal barrier proteins were assessed via immunohistochemistry. Briefly, paraffin-embedded sections underwent deparaffinization, rehydration, and heat-induced antigen retrieval in Tris-EDTA buffer under pressurized conditions. Endogenous peroxidase activity was quenched with 3% hydrogen peroxide, and non-specific binding was blocked with 5% BSA. Sections were sequentially probed with primary antibodies against ZO-1 (Sigma-Aldrich, St. Louis, MO, USA, Cat# SAB5700645, 1:200), claudin-1 (Sigma-Aldrich, St. Louis, MO, USA, Cat# SAB4503546, 1:200), and occludin (Sigma-Aldrich, St. Louis, MO, USA, Cat# SAB4200593, 1:200) followed by species-matched secondary antibodies. Immunoreactivity was visualized using DAB chromogen, and nuclei were counterstained with hematoxylin. Images were acquired under light microscopy, and positive staining areas were quantified using ImageJ software [27].

### 2.9. Immunofluorescence Detection of HCAR2

HCAR2 protein abundance in hepatic and intestinal tissues was examined by immunofluorescence. Following deparaffinization, antigen retrieval, and blocking steps as detailed in Section 2.8, Sections were incubated with anti-HCAR2 primary antibody (Sigma-Aldrich, St. Louis, MO, USA, Cat# SAB4301084, 1:200) for 1 h at ambient temperature. After TBS rinses, fluorophore-conjugated secondary antibody was applied for 30 min in darkness. Sections were subsequently dehydrated, cleared, mounted, and visualized under a fluorescence microscope. Quantitative analysis of positive staining was performed using ImageJ 1.53t (NIH, Bethesda, MD, USA) [28].

### 2.10. RT-qPCR Detection

Total RNA was isolated from liver specimens using TRNzol Universal reagent (Tiangen Biotech (Beijing) Co., Ltd., Beijing, China). Tissue (approximately 0.03 g) was homogenized in the reagent under chilled conditions, followed by chloroform-mediated phase separation and isopropanol precipitation. The RNA precipitate was washed with 75% ethanol, air-dried, and resuspended in nuclease-free water. RNA integrity was verified by agarose gel electrophoresis, and concentration plus purity were assessed spectrophotometrically. Complementary DNA was synthesized from 1 μg of total RNA using ReverTra Ace qPCR RT Master Mix (Toyobo (Shanghai) Biotechnology Co., Ltd., Shanghai, China). Quantitative real-time PCR was conducted with SYBR Green Realtime PCR Master Mix and gene-specific primers (Sangon Biotech (Shanghai, China) Co., Ltd., Shanghai, China; sequences provided in Table 1) on a real-time thermal cycler [29].

### 2.11. Western Blot Detection of Liver Protein Levels

Total protein was extracted from liver tissue using RIPA buffer supplemented with protease/phosphatase inhibitors (Beijing Solarbio Science & Technology Co., Ltd., Beijing, China). Protein concentrations were determined via BCA assay. Denatured samples were resolved by SDS-PAGE and electrotransferred onto methanol-activated PVDF membranes. After blocking, membranes were probed overnight at 4 °C with primary antibodies against NLRP3 (Sigma-Aldrich, Cat# SAB5700723, 1:1000), caspase-1 (Sigma-Aldrich, St. Louis, MO, USA, Cat# C4851, 1:1000), caspase-1 p20 (Sigma-Aldrich, St. Louis, MO, USA, Cat# SAB4503272, 1:1000), and β-actin (Sigma-Aldrich, St. Louis, MO, USA, Cat# A5441, 1:5000). Following TBST washes, HRP-conjugated secondary antibodies (Absin Bioscience Inc., Shanghai, China) were applied for 1 h. Immunoreactive bands were detected using SuperSignal chemiluminescent substrate (Thermo Fisher Scientific (Beijing, China) Co., Ltd., Shanghai, China) and quantified densitometrically withImageJ 1.53t (National Institutes of Health, Bethesda, MD, USA) [30].

### 2.12. 16S rRNA Microbial Diversity Sequencing Analysis

Intestinal contents were collected post-intervention for microbial community profiling [31]. The 16S rRNA sequencing and subsequent bioinformatic analysis were performed by Shanghai Majorbio Bio-Pharm Technology Co., Ltd. (Shanghai, China). Total genomic DNA was extracted using the MolPure Stool DNA Kit (Yeasen Biotechnology, Shanghai, China; Cat# 18800ES50) according to the manufacturer’s instructions. DNA concentration and purity were assessed via Qubit 2.0 and NanoDrop, with integrity confirmed by agarose gel electrophoresis. The V3-V4 hypervariable region of the 16S rRNA gene was amplified using primers 338F/806R. Purified amplicons were pooled in equimolar amounts and paired-end sequenced on the Illumina NovaSeq PE250 platform. The sequencing generated an average of 64,957 raw reads per sample (total 1,948,715 raw reads). After quality filtering, an average of 63,129 high-quality reads per sample (total 1,893,865 sequences; average length 422 bp) were obtained for subsequent bioinformatic analysis [32].

### 2.13. Untargeted Metabolomics

Intestinal content samples were submitted to Shanghai Majorbio Bio-pharm Technology Co., Ltd. for untargeted metabolomic profiling. Downstream statistical analyses were conducted on the Majorbio Cloud Platform (https://www.majorbio.com, accessed on 15 May 2026). The specific methods are as follows: Intestinal content samples (20 ± 5 mg) were homogenized with a 6 mm grinding bead in 400 µL of extraction solution (methanol: water = 4:1, *v*/*v*) containing four internal standards (0.02 mg/mL L-2-chlorophenylalanine, etc.) using a frozen tissue grinder (Wonbio-96c, Shanghai Wanbo Biotechnology, Shanghai, China) for 6 min (−10 °C, 50 Hz), followed by low-temperature ultrasonic extraction for 30 min (5 °C, 40 kHz). After standing at −20 °C for 30 min and centrifugation at 13,000× *g* for 15 min (4 °C), the supernatant was collected for LC-MS/MS analysis. A pooled quality control (QC) sample was prepared by mixing equal volumes of all supernatants. LC-MS/MS analysis was performed on a Thermo Scientific UHPLC-Orbitrap Exploris 240 system (Thermo Fisher Scientific, Waltham, MA, USA) equipped with an ACQUITY UPLC HSS T3 column (100 mm × 2.1 mm i.d., 1.8 µm; Waters, Milford, MA, USA). The mobile phases were solvent A (95% water, 5% acetonitrile, 0.1% formic acid) and solvent B (47.5% acetonitrile, 47.5% isopropanol, 5% water, 0.1% formic acid). The injection volume was 3 µL, the flow rate was 0.40 mL/min, and the column temperature was 40 °C. MS data were acquired in both positive and negative ion modes (*m*/*z* 70–1050) with spray voltages of 3400 V and −3000 V, respectively. The capillary and heater temperatures were 320 °C and 350 °C, and the normalized collision energy was set at 20, 40, and 60 V. Raw data were processed using Progenesis QI v3.0 (Waters Corporation, Milford, MA, USA) for peak alignment and identification against the HMDB, Metlin, and self-built Majorbio databases (mass error < 10 ppm). Data matrices were uploaded to the Majorbio Cloud Platform, where variables with QC RSD > 30% were excluded, and differential metabolites were screened based on VIP > 1 (OPLS-DA) and *p* < 0.05 (Student’s *t*-test).

### 2.14. Statistical Analysis

Data analysis was performed using SPSS 26.0 and GraphPad Prism 10. Results are presented as mean ± standard deviation. For each endpoint, normality was assessed by the Shapiro–Wilk test and homogeneity of variances by Levene’s test. When both assumptions were satisfied (*p* > 0.05), one-way ANOVA followed by Tukey’s post hoc test was used, which controls the family-wise error rate across all pairwise comparisons within that endpoint. When either assumption was violated (*p* < 0.05), the Kruskal–Wallis test followed by Dunn’s post hoc test with Benjamini–Hochberg adjustment was used. Specifically, one-way ANOVA with Tukey’s post hoc test was applied to serum lipid parameters, serum inflammatory cytokines, hepatic injury markers, hepatic and intestinal mRNA expression levels, and immunohistochemistry and immunofluorescence quantification; the Kruskal–Wallis test with Dunn’s post hoc test was applied to alpha diversity indices; PERMANOVA was used for beta diversity analysis; and for untargeted metabolomics, differential metabolites were screened using VIP > 1 (OPLS-DA) and *p* < 0.05 (Student’s *t*-test), with KEGG pathway enrichment *p* values adjusted by the Benjamini–Hochberg procedure. Correlations were assessed by Spearman’s rank test. Statistical significance was defined as *p* < 0.05.

## 3. Results

### 3.1. The Effect of Niacin Combined with B. animalis subsp. lactis F1-7 on Lipid Metabolism in Mice

**Figure 1:**
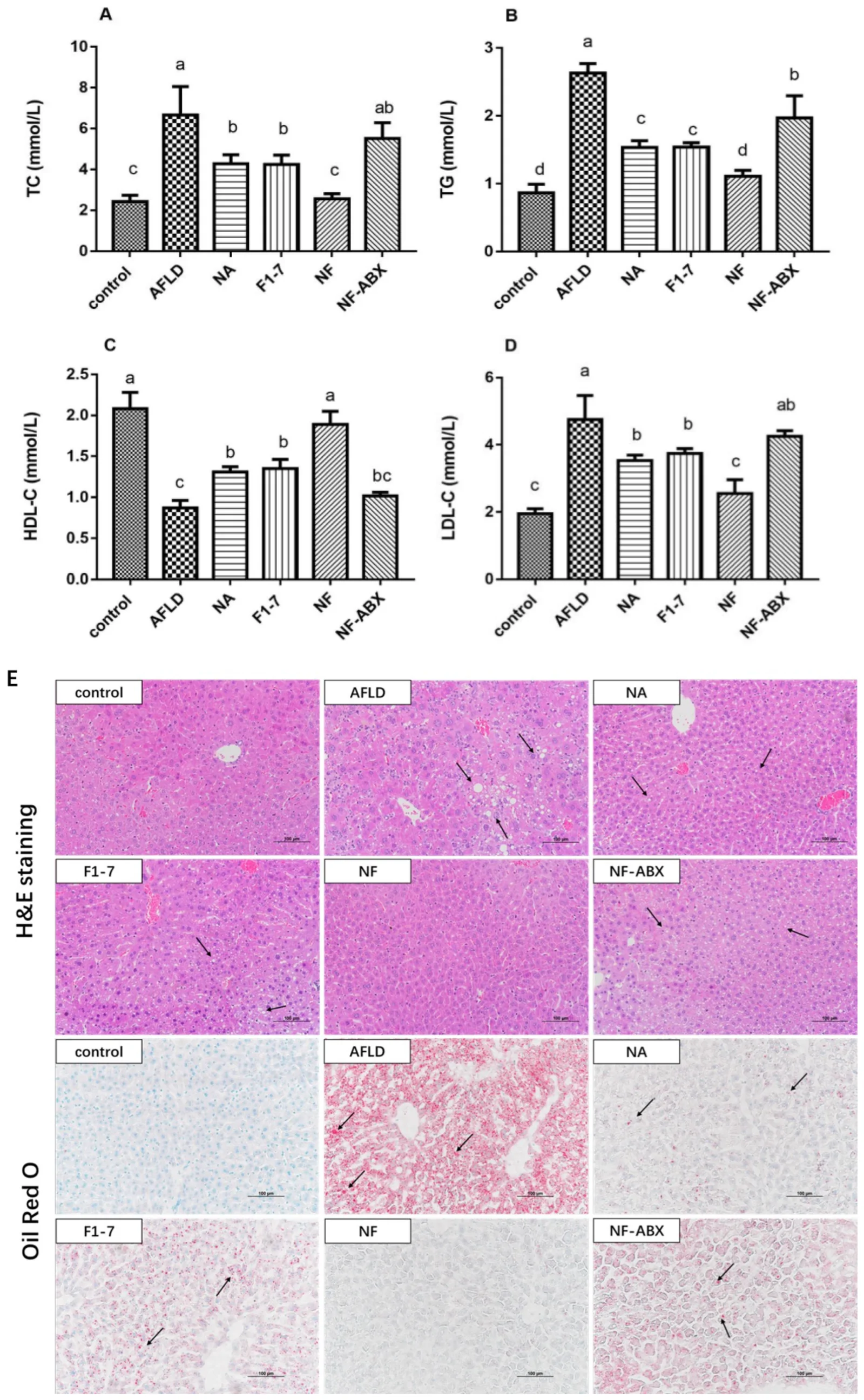
Impact of niacin combined with *B. animalis* subsp. *lactis* F1-7 on murine lipid metabolism (*n* = 6 per group for serum lipid assays; *n* = 3 per group for histology). (**A**) TC levels; (**B**) TG levels; (**C**) HDL-C levels; (**D**) LDL-C levels. Different letters indicate statistically significant differences, *p* < 0.05; (**E**) H&E staining and Oil Red O staining of mouse liver tissue. Arrows indicate lipid vacuoles and inflammatory cell infiltration in H&E-stained sections, and lipid droplets in Oil Red O-stained sections.Scale bar: 100 μm.

To assess the lipid-modulating effect of the combination therapy, serum lipid levels were determined (Figure 1A–D). Relative to the Control group, the AFLD group displayed significantly higher TC, TG, and LDL-C, along with a marked decrease in HDL-C (*p* < 0.05). Either niacin (NA) or F1-7 alone improved these parameters, but no significant difference was observed between the two monotherapy groups. The combined intervention (NF) resulted in significantly greater improvements than all other intervention groups (*p* < 0.05), with TC and TG decreased by 61.69% and 57.79%, respectively, and the greatest changes in HDL-C and LDL-C. TC and TG in the NF group no longer differed significantly from the Control group (*p* > 0.05). After antibiotic treatment, the NF-ABX group showed significantly weaker improvements than the NF group (HDL-C increased by only 16.67% and LDL-C decreased by only 10.71%). These results indicate that the combined intervention effectively improves serum lipid profiles in alcoholic fatty liver mice, with superior effects to either monotherapy, and that this effect is dependent on an intact gut microbiota. Liver histology (Figure 1E) revealed normal architecture in the Control group. The AFLD group exhibited disorganized hepatic cords, obvious lipid vacuoles in hepatocytes, extensive inflammatory cell infiltration, and abundant deep-stained lipid droplets on Oil Red O staining. All intervention groups showed improvements, with the NF group displaying relatively orderly hepatic cords, the mildest lipid vacuoles and inflammatory infiltration, and the least lipid droplet accumulation. The NF-ABX group still exhibited some lipid vacuoles and marked lipid droplet accumulation. These findings indicate that the combined intervention significantly alleviates alcohol-induced hepatic inflammation and lipid accumulation, and that antibiotic treatment attenuates its hepatoprotective effects.

### 3.2. Effect of Niacin Combined with B. animalis subsp. lactis F1-7 on Inflammatory Responses in Mice

**Figure 2:**
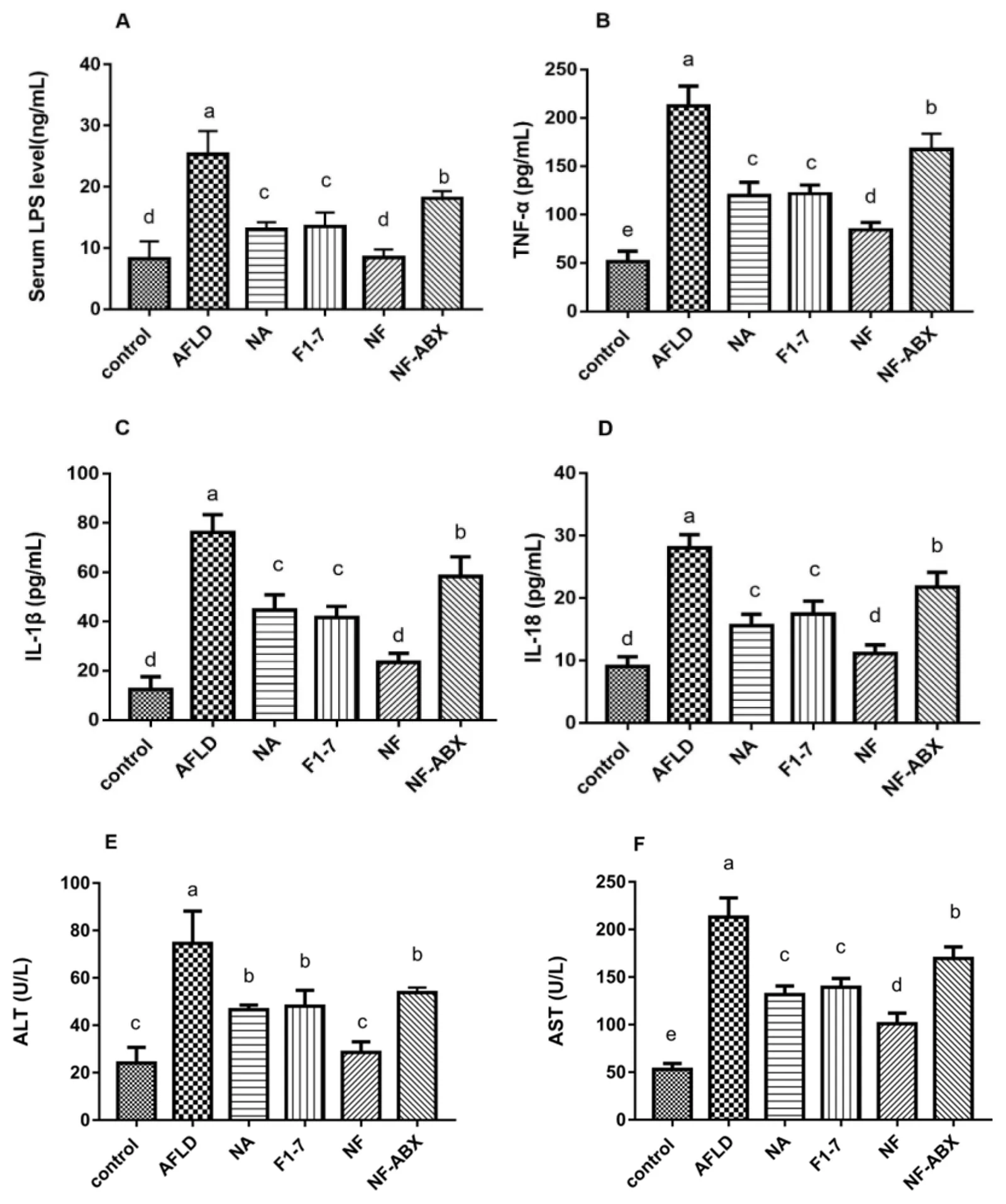
Effect of niacin combined with *B. animalis* subsp. *lactis* F1-7 on inflammatory responses in mice (*n* = 6). (**A**) Serum LPS levels; (**B**) Serum TNF-α levels; (**C**) Serum IL-1β levels; (**D**) Serum IL-18 levels; (**E**) Hepatic ALT levels; (**F**) Hepatic AST levels. Different lowercase letters indicate statistically significant differences, *p* < 0.05.

Compared with the AFLD group, serum LPS levels decreased by 66.49% in the NF group, exceeding reductions seen with NA (48.26%) or F1-7 (46.53%) monotherapy (*p* < 0.05). The NF-ABX group showed only a 28.31% decline (Figure 2A). Similarly, TNF-α, IL-1β, and IL-18 levels in the NF group dropped by 60.34%, 69.25%, and 60.35%, respectively, significantly greater than the 41–45% reductions observed with single-agent treatments. Meanwhile, NF-ABX exhibited ≤30% suppression (Figure 2B–D). Hepatic ALT and AST activities were reduced by 61.74% and 52.58% in the NF group versus 35–38% in monotherapy groups. In contrast, NF-ABX lowered ALT and AST by only 27.85% and 20.42% (Figure 2E,F). These results confirm that the combined intervention markedly ameliorates alcohol-induced inflammatory and hepatic damage, and that this protection is largely abrogated by gut microbiota depletion.

### 3.3. Regulatory Effect of Niacin Combined with B. animalis subsp. lactis F1-7 on Key Inflammatory Targets in Mice

**Figure 3:**
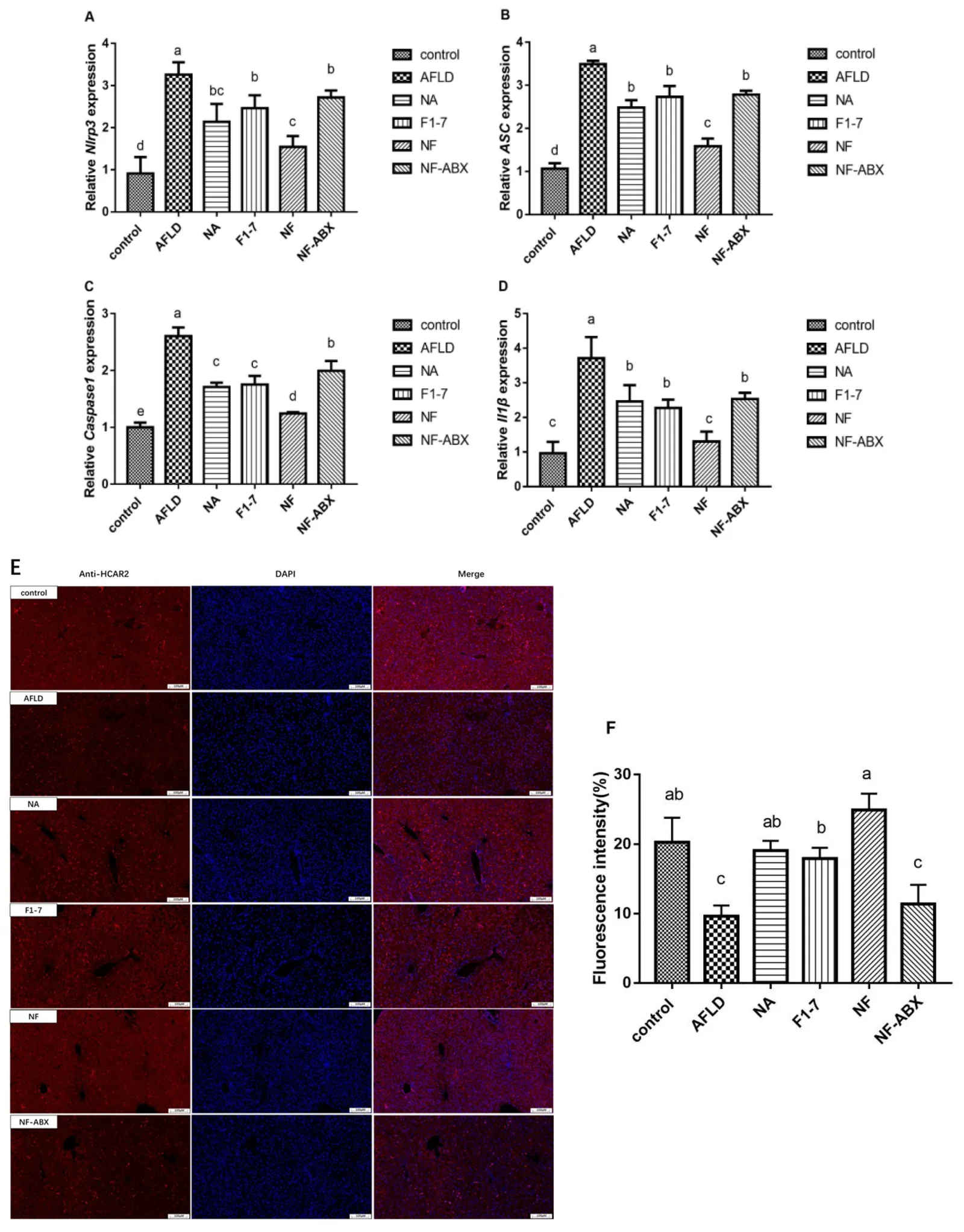

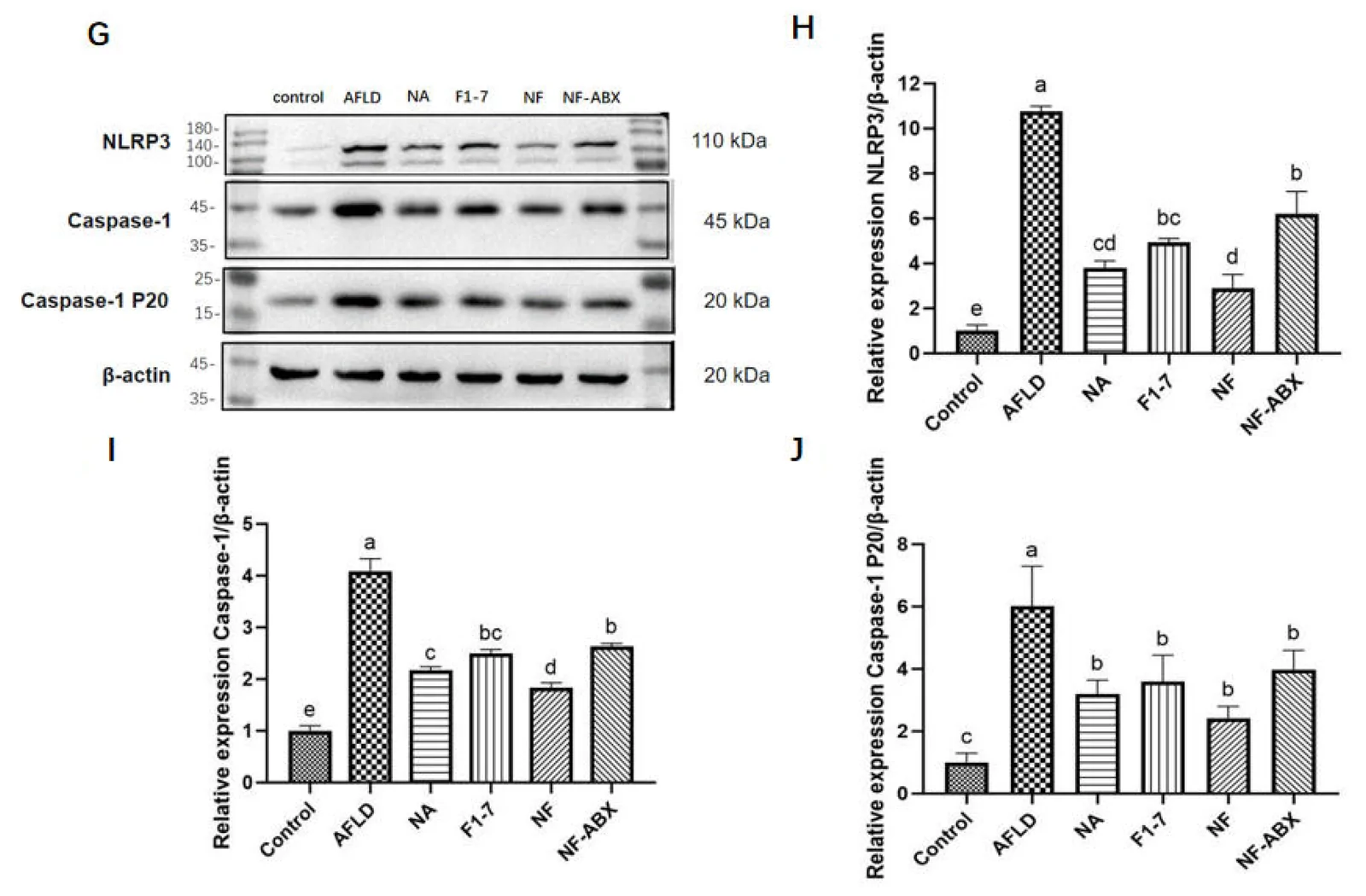
Effect of niacin combined with *B. animalis* subsp. *lactis* F1-7 on the expression levels related to hepatic inflammation in mice (*n* = 4). (**A**) Hepatic *NLRP3* gene expression; (**B**) hepatic *ASC* gene expression; (**C**) hepatic *Caspase-1* gene expression; (**D**) hepatic *IL-1β* gene expression; (**E**) immunofluorescence staining of hepatic HCAR2 protein. Scale bar: 100 μm. (**F**) Fluorescence intensity of hepatic HCAR2 protein; (**G**) Western blot results of NLRP3, Caspase-1, and Caspase-1 P20 proteins; (**H**) relative expression level of hepatic NLRP3 protein; (**I**) relative expression level of hepatic Caspase-1 protein; (**J**) relative expression level of hepatic Caspase-1 P20 protein. Different lowercase letters indicate statistically significant differences, *p* < 0.05.

RT-qPCR analysis revealed that the NF group exhibited the most pronounced suppression of hepatic *NLRP3*, *ASC*, *Caspase-1*, and *IL-1β* mRNA expression, with reductions of 48.58%, 54.96%, 52.17%, and 63.77%, respectively, relative to the AFLD group. These effects were markedly attenuated in the NF-ABX group, where reductions ranged from only 19.45% to 26.69% (Figure 3A–D). Immunofluorescence staining demonstrated that HCAR2 protein levels were significantly elevated in the NA, F1-7, and NF groups compared with AFLD controls (*p* < 0.05), with the NF group showing the greatest increase. No appreciable change was observed in NF-ABX mice (Figure 3E,F). Western blotting further confirmed that NLRP3, Caspase-1, and Caspase-1 p20 protein abundances were substantially reduced in all intervention groups versus AFLD (*p* < 0.05), with NF achieving the most significant attenuation and NF-ABX displaying the least improvement (Figure 3G–J). Collectively, these data indicate that the combined intervention alleviates hepatic inflammation in AFLD mice through microbiota-dependent modulation of the HCAR2–NLRP3 signaling pathway.

### 3.4. Effect of Niacin Combined with B. animalis subsp. lactis F1-7 on Intestinal Permeability in Mice

**Figure 4:**
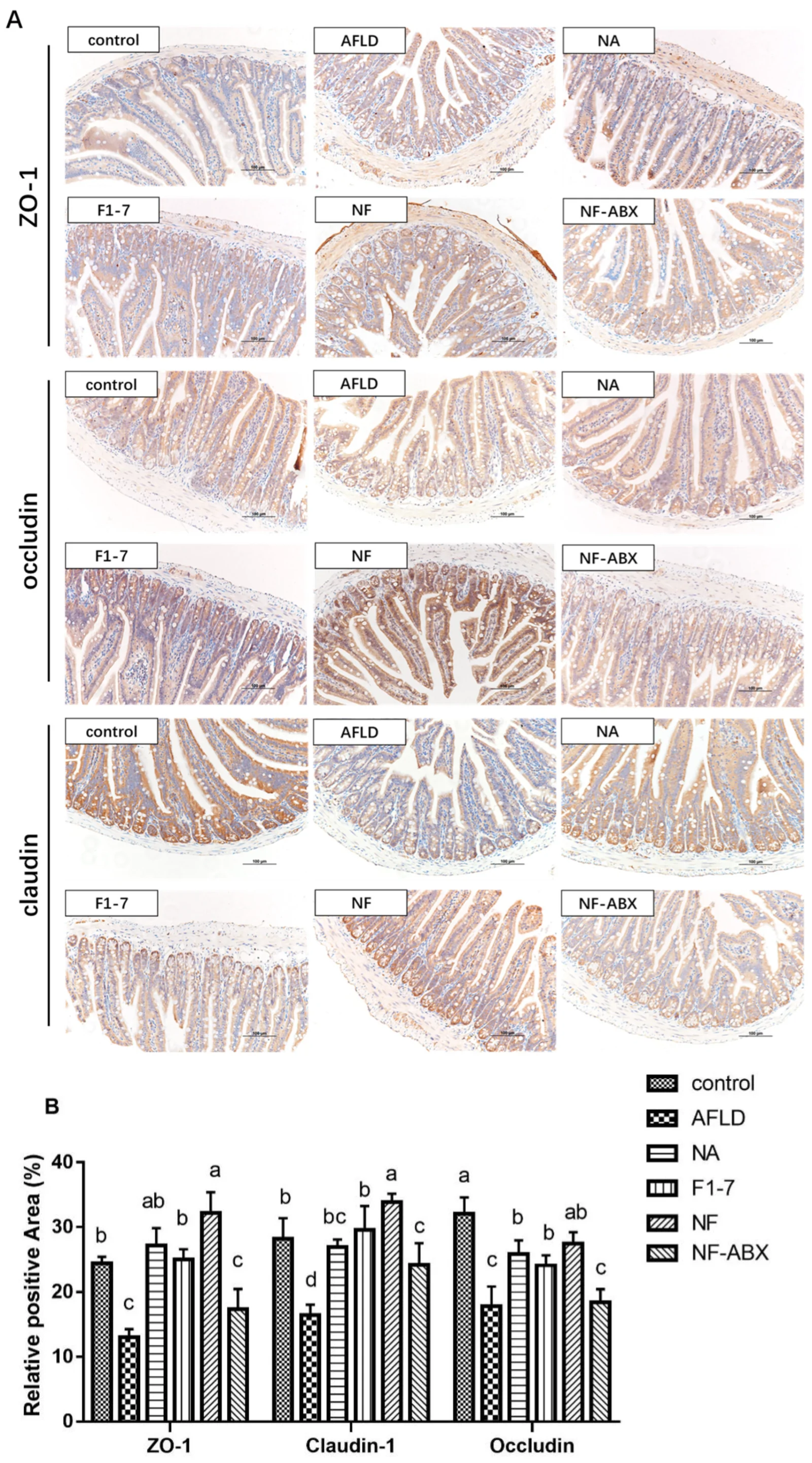
Effect of niacin combined with *B. animalis* subsp. *lactis* F1-7 on intestinal permeability in mice (*n* = 4). (**A**) Immunohistochemical staining of intestinal permeability-related proteins; (**B**) gray values of intestinal permeability-related protein immunohistochemistry. Scale bar: 100 μm. Different lowercase letters indicate statistically significant differences, *p* < 0.05.

Immunohistochemical analysis of tight junction proteins revealed that ZO-1, claudin-1, and occludin expression were significantly reduced in the AFLD group relative to controls (*p* < 0.05). While NA, F1-7, and NF interventions all elevated these barrier proteins compared with AFLD (*p* < 0.05), the NF group exhibited the most pronounced restoration, with claudin-1 and occludin levels significantly exceeding those of other intervention groups (*p* < 0.05). Notably, the NF-ABX group displayed minimal improvement across all three markers, indicating that antibiotic-mediated microbiota depletion largely abrogated the barrier-protective effect (Figure 4). These findings demonstrate that the combined intervention effectively attenuates alcohol-induced intestinal hyperpermeability, and that this protection is critically dependent on an intact gut microbiota.

### 3.5. Regulatory Effect of Niacin Combined with B. animalis subsp. lactis F1-7 on the Intestinal HCAR2/NLRP3/Caspase-1 Pathway in Mice

**Figure 5:**
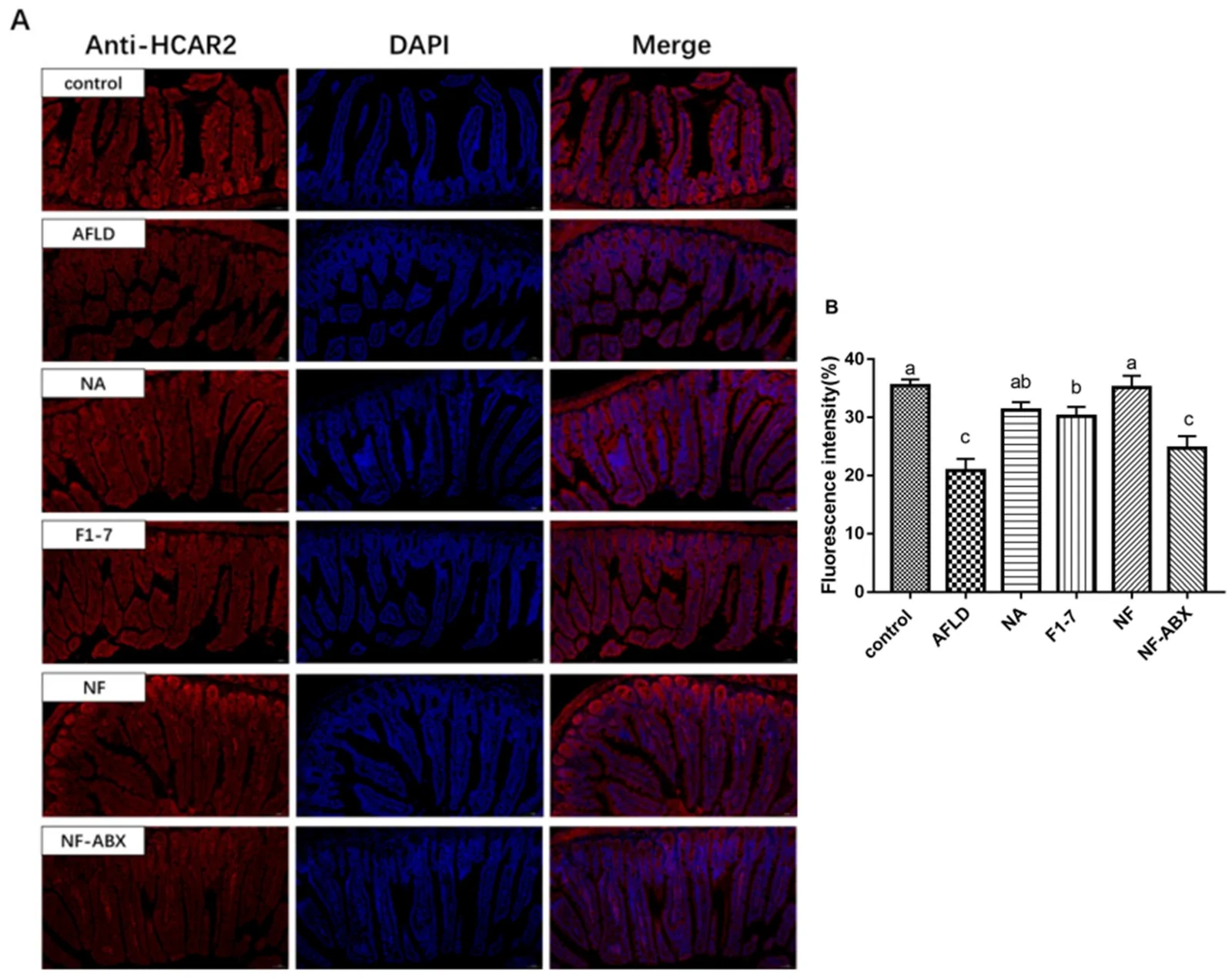

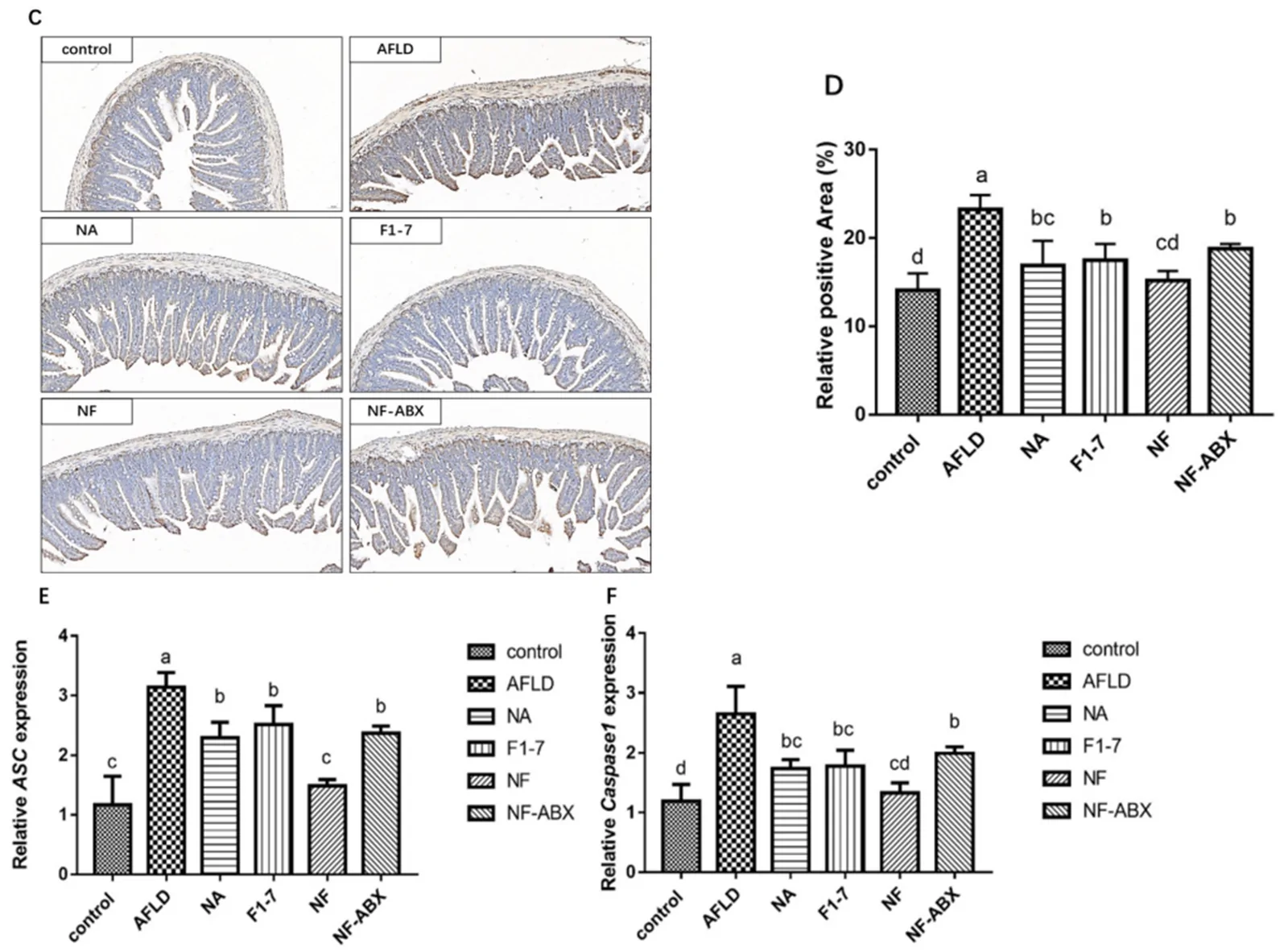
Regulatory effect of niacin combined with *B. animalis* subsp. *lactis* F1-7 on key targets of intestinal inflammation in mice (*n* = 4). (**A**) Immunofluorescence staining of intestinal HCAR2 protein; (**B**) fluorescence intensity of intestinal HCAR2 protein; (**C**) immunohistochemical staining of intestinal NLRP3 protein; (**D**) gray value of intestinal NLRP3 protein immunohistochemistry; (**E**) intestinal *ASC* gene expression; (**F**) intestinal *Caspase-1* gene expression. Scale bar: 100 μm. Different lowercase letters indicate statistically significant differences, *p* < 0.05.

Immunofluorescence analysis revealed that intestinal HCAR2 protein levels were significantly diminished in the AFLD group relative to controls (*p* < 0.05). Intervention with NA, F1-7, or NF markedly restored HCAR2 fluorescence intensity (*p* < 0.05), with NF eliciting the greatest upregulation and NA surpassing F1-7. No appreciable recovery was detected in the NF-ABX group (Figure 5A,B). Immunohistochemical staining demonstrated that NLRP3 abundance was reduced in all intervention groups compared with AFLD (*p* < 0.05), with the NF group exhibiting the lowest positive area among all intervention cohorts (Figure 5C,D). Intestinal *ASC* and *Caspase-1* mRNA levels were elevated in AFLD mice and were significantly suppressed by all interventions (*p* < 0.05). The NF group achieved the most pronounced reductions (52.79% and 49.64%, respectively), whereas NF-ABX attenuated these transcripts by only 23.23% and 24.71% (Figure 5E,F). These data indicate that the combined intervention robustly downregulates intestinal inflammatory gene expression in a microbiota-dependent manner.

### 3.6. Effect of Niacin Combined with B. animalis subsp. lactis F1-7 on the Gut Microbiota in Mice

**Figure 6:**
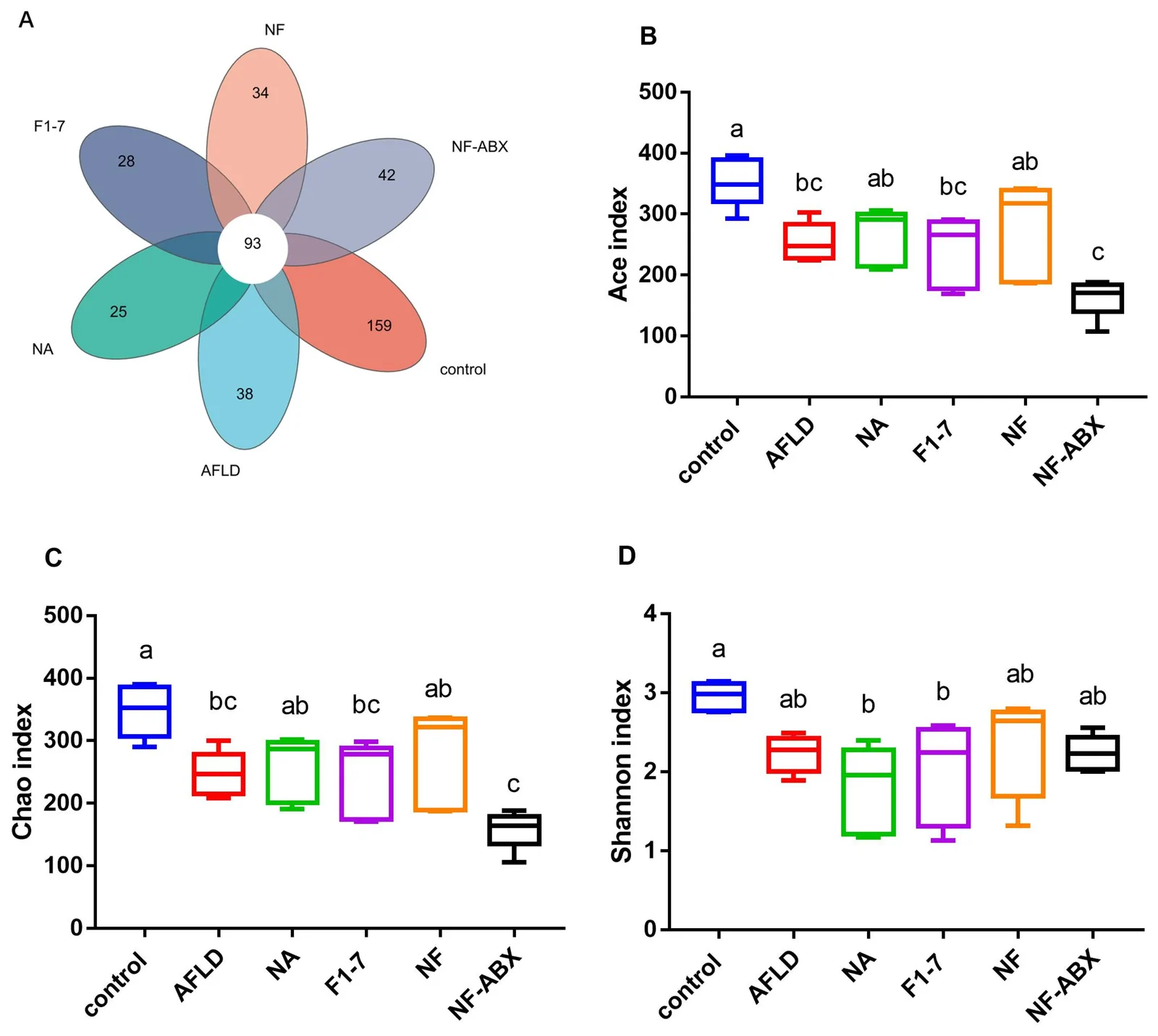

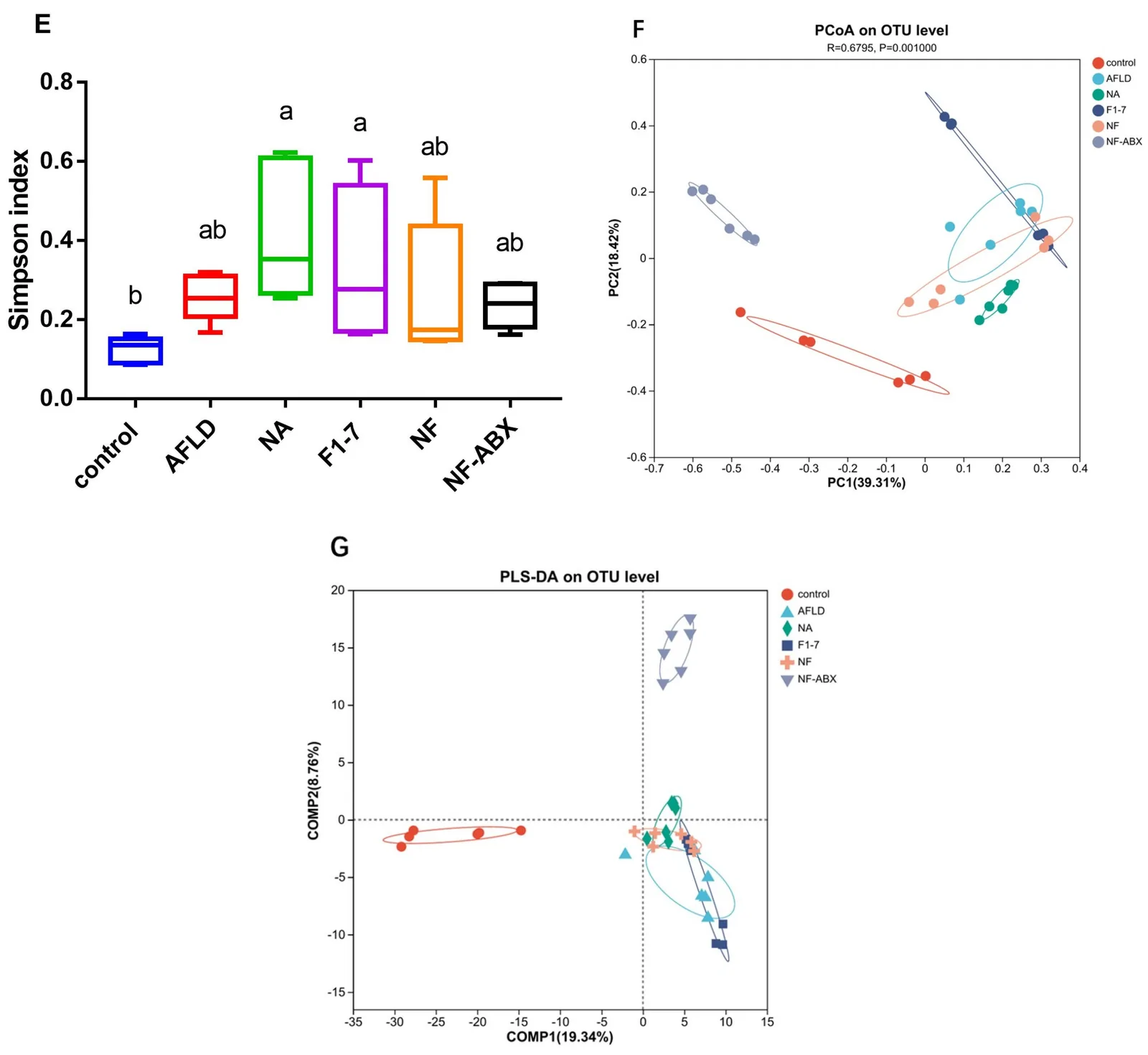
Effect of niacin combined with *B. animalis* subsp. *lactis* F1-7 on the gut microbiota in mice (*n* = 6). (**A**) OTU distribution; (**B**) Ace index; (**C**) Chao index; (**D**) Shannon index; (**E**) Simpson index; (**F**) PCoA plot based on OTU level; (**G**) PLS-DA plot based on OTU level. Different letters indicate statistically significant differences, *p* < 0.05.

The 16S rRNA sequencing of intestinal contents identified 93 shared OTUs across all six groups, with the control group harboring the highest number of unique OTUs (159) (Figure 6A). Alpha diversity analysis revealed that alcohol exposure (AFLD) significantly reduced species richness, as indicated by the Ace and Chao indices, compared with controls (*p* < 0.05). However, the Shannon and Simpson indices, which reflect both richness and evenness, did not show statistically significant differences between the AFLD and Control groups (*p* > 0.05) (Figure 6B–E). Ace and Chao values in the NF group did not differ from those of controls, indicating partial restoration of richness. In contrast, the NF-ABX group exhibited markedly reduced alpha diversity compared with NF, underscoring the detrimental impact of antibiotic pretreatment. Beta diversity assessment via PCoA (PC1: 39.31%, PC2: 18.42%) and PLS-DA demonstrated distinct clustering of all intervention groups away from controls, with the NF-ABX group displaying the most pronounced divergence, confirming that antibiotic exposure substantially remodels gut microbial community structure (Figure 6F,G).

**Figure 7:**
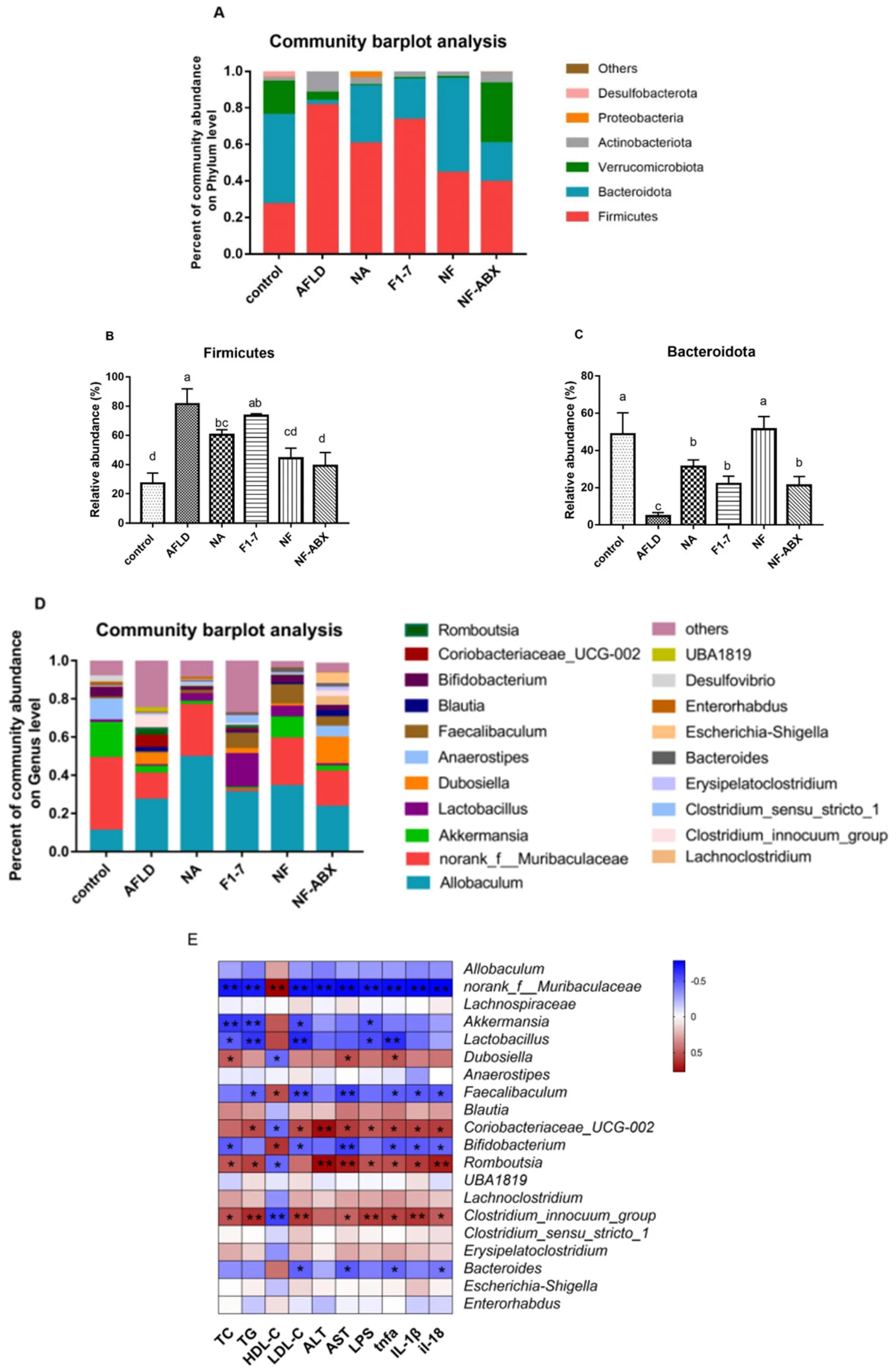
Modulation of gut microbiota composition by niacin combined with *B. animalis* subsp. *lactis* F1-7. (**A**) Phylum-level relative abundance. (**B**) Firmicutes abundance. (**C**) Bacteroidota abundance. (**D**) Genus-level relative abundance. (**E**) Spearman correlations between differentially abundant genera and serum inflammatory/lipid parameters. Bars labeled with distinct lowercase letters denote statistically significant differences, *p* < 0.05. In the heatmap, * *p* < 0.05 and ** *p* < 0.01.

Phylum-level profiling revealed that the gut microbiota was dominated by Firmicutes, Bacteroidota, Verrucomicrobiota, Actinobacteriota, Proteobacteria, and Desulfobacterota (Figure 7A–C). Relative to controls, AFLD mice exhibited elevated Firmicutes and reduced Bacteroidota abundance (*p* < 0.05). The NA, NF, and NF-ABX interventions significantly reversed this imbalance, with the NF group showing the greatest enrichment of Bacteroidota (*p* < 0.05). Among the top 20 abundant genera, *Allobaculum*, *norank_f_Muribaculaceae*, *Lactobacillus*, *Faecalibaculum*, *Bifidobacterium*, and *Bacteroides* were increased, whereas *Dubosiella*, *Blautia*, Coriobacteriaceae_UCG-002, *Romboutsia*, *UBA1819*, and *Clostridium_innocuum_group* were decreased in the AFLD group compared with the control group (Figure 7D). Spearman correlation analysis (Figure 7E) revealed that *norank_f_Muribaculaceae*, *Faecalibaculum*, and *Bifidobacterium* were positively correlated with HDL-C but negatively correlated with most other lipid, liver injury, and inflammatory markers (*p* < 0.05), while *Romboutsia*, Coriobacteriaceae_UCG-002, *Clostridium_innocuum_ group*, and *Dubosiella* showed positive correlations with most of these parameters (*p* < 0.05).

### 3.7. Effect of Niacin Combined with B. animalis subsp. lactis F1-7 on Intestinal Metabolites in Mice

**Figure 8:**
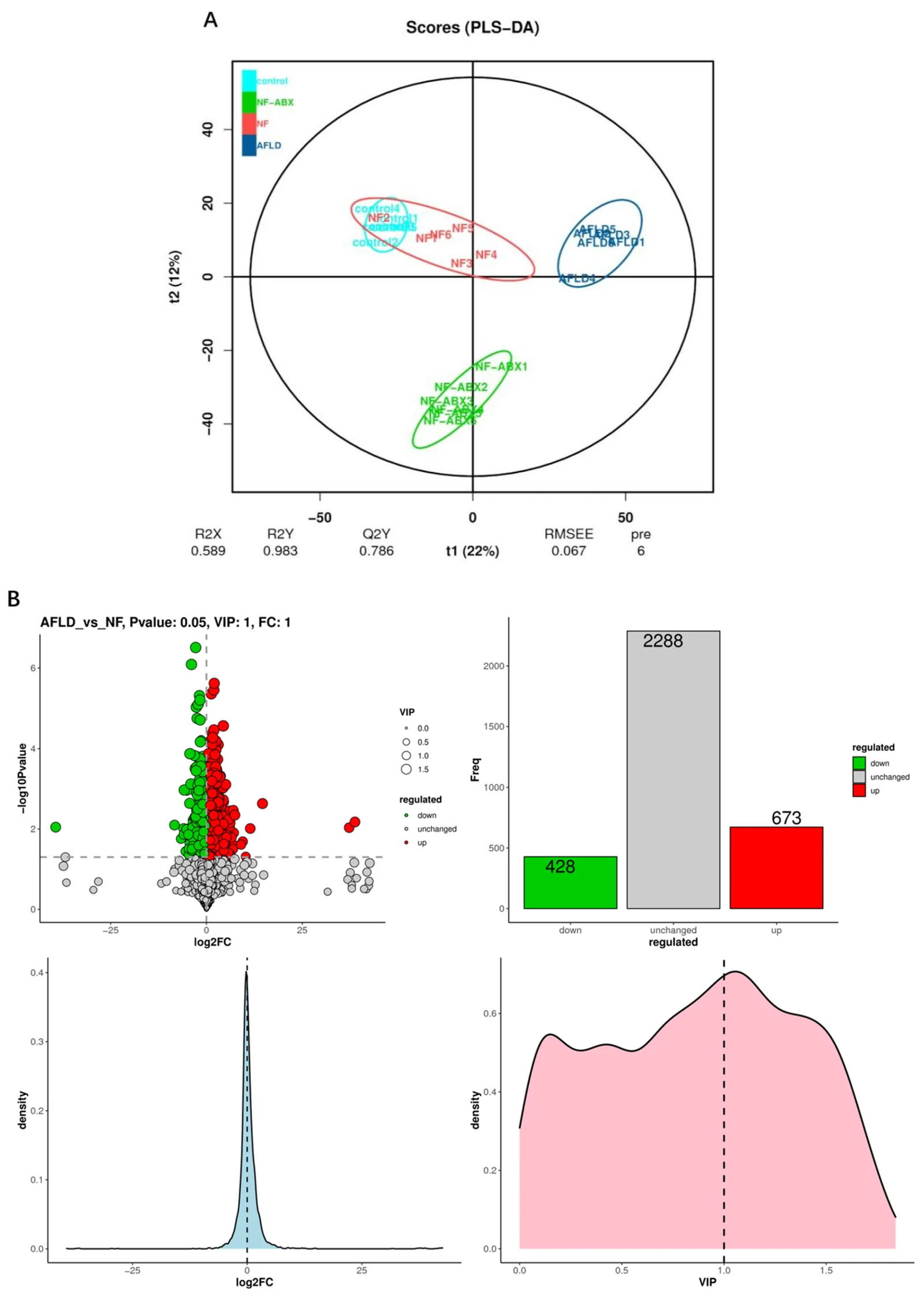

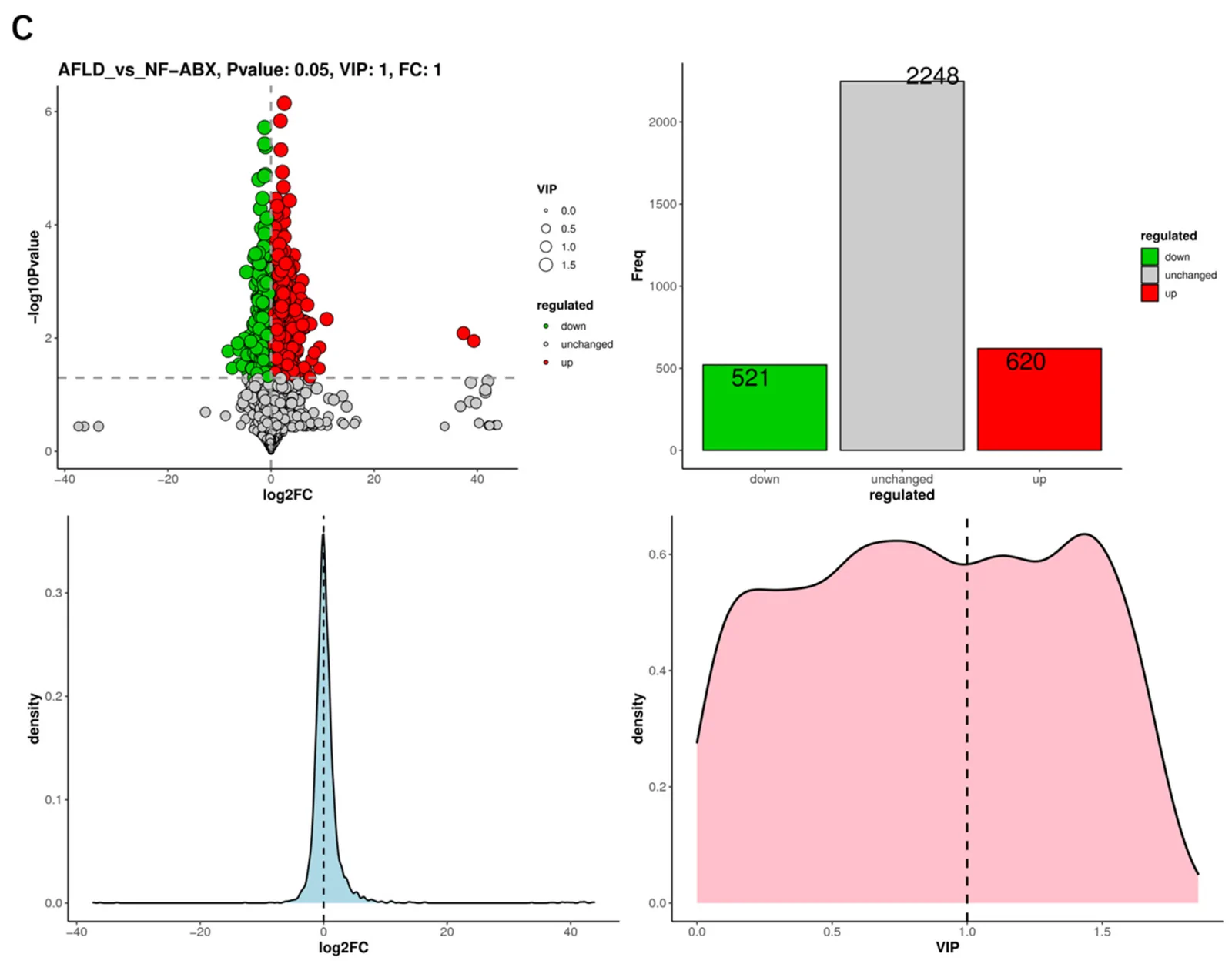
Overall profile of intestinal metabolites and intergroup differential expression analysis (*n* = 6). (**A**) PLS-DA plot; (**B**) Overview of differential metabolites between the AFLD and NF groups; (**C**) Overview of differential metabolites between the AFLD and NF-ABX groups. In both (**B**,**C**), the horizontal dashed line in the volcano plot (top left) indicates the significance threshold (*p* = 0.05; −log10 *p* = 1.3), and the vertical dashed lines indicate the fold change thresholds (|log2FC| = 1). In the density plots of log2FC (bottom left) and VIP score (bottom right), the vertical dashed lines indicate the thresholds of log2FC = 0 and VIP = 1, respectively.

The results of the PLS-DA model are shown in Figure 8A. The model achieved a fit parameter (R^2^) of 0.983 and a predictive parameter (Q^2^) of 0.786, indicating that the model was stable and reliable without overfitting, and could effectively explain and predict metabolic differences among samples. Differential intestinal metabolites were screened between AFLD and NF or NF-ABX groups. Relative to AFLD, the NF group exhibited 1101 significantly altered metabolites (428 downregulated, 673 upregulated), whereas the NF-ABX group showed 1141 differential metabolites (521 downregulated, 620 upregulated) (Figure 8B,C).

**Figure 9:**
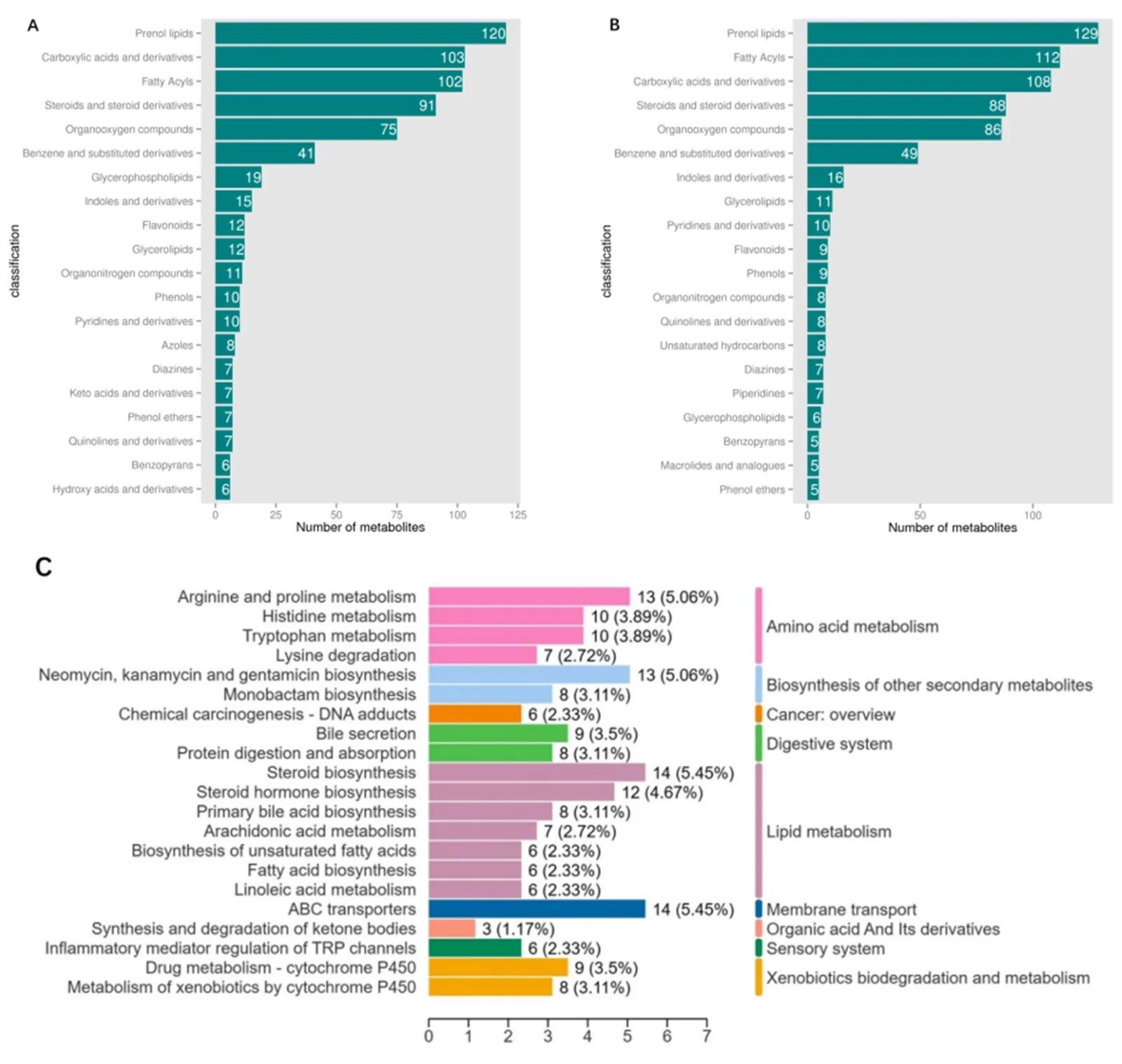

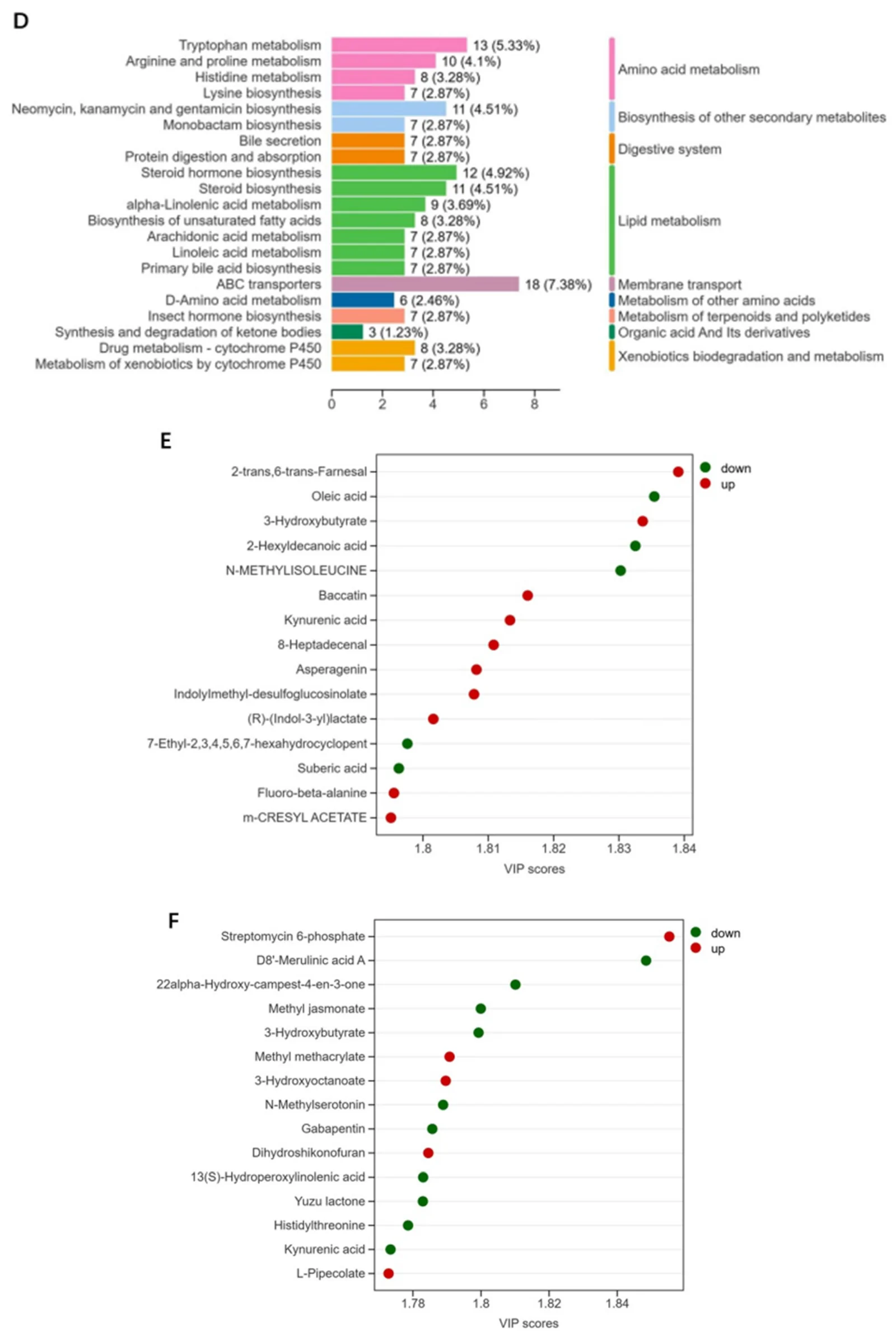
Chemical classification, KEGG enrichment, and VIP ranking of differential intestinal metabolites (*n* = 6). (**A**) HMDB chemical subclass distribution of differential metabolites between the AFLD and NF groups. (**B**) HMDB chemical subclass distribution of differential metabolites between the NF and NF-ABX groups. (**C**) KEGG pathway enrichment of differential metabolites in the NF group. (**D**) KEGG pathway enrichment in the NF-ABX group. (**E**) VIP score plot of top-ranked metabolites comparing AFLD versus NF. (**F**) VIP score plot comparing NF versus NF-ABX.

The differential metabolites were classified according to their chemical categories using the HMDB database. As shown in Figure 9A,B, these metabolites were widely distributed across multiple chemical subclasses. In the comparison between the AFLD and NF groups, 120 prenol lipids, 103 carboxylic acids and derivatives, 102 fatty acyls, and 91 steroids and steroid derivatives were identified. In the comparison between the AFLD and NF-ABX groups, 129 prenol lipids, 112 fatty acyls, 108 carboxylic acids and derivatives, and 88 steroids and steroid derivatives were identified. These results indicated that the changes in the intestinal metabolite profiles in both the NF and NF-ABX groups were mainly concentrated in lipids and organic acids. KEGG pathway enrichment analysis indicated that lipid metabolism pathways, including steroid biosynthesis, primary bile acid biosynthesis, fatty acid metabolism, and ketone body synthesis/degradation, were prominently altered in the NF group (Figure 9C,D). Notably, the synthesis and degradation of ketone bodies pathway was also enriched, in which the core metabolite β-hydroxybutyrate serves as a key node linking lipid metabolism to host energy and immune regulation. These findings suggest that the combined intervention reshapes the mouse lipid metabolism network and creates a favorable metabolic environment for the production of ketone bodies by modulating bile acid and steroid metabolism. Analysis of the top 15 differential metabolites ranked by variable importance in projection (VIP) score is shown in Figure 9E,F. In the comparison between the AFLD and NF groups, β-hydroxybutyrate, the core product of ketone body metabolism, ranked third in VIP score and showed a significantly increasing trend in the NF group. In contrast, in the NF-ABX group, the relative abundance of β-hydroxybutyrate showed a significantly decreasing trend. These results indicate that the combined intervention specifically activates the ketone body metabolic pathway centered on β-hydroxybutyrate through modulation of the gut microbiota. After antibiotic depletion of the gut microbiota, the level of β-hydroxybutyrate decreased, demonstrating that its intestinal synthesis depends on an intact gut microbiota structure.

**Figure 10:**
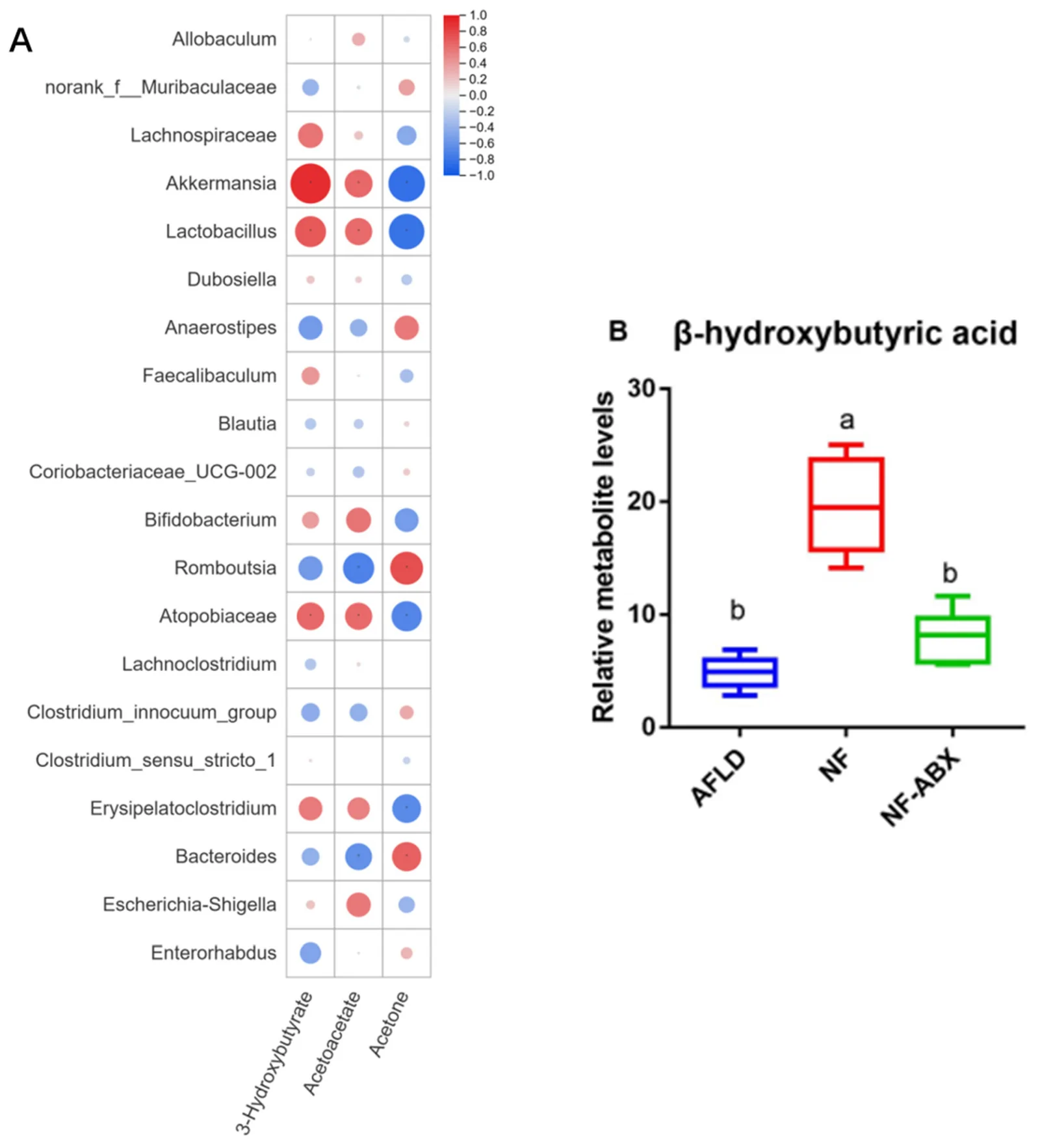
Correlation between differential bacterial genera and ketone body metabolites and intestinal β-hydroxybutyrate abundance among groups (*n* = 6). (**A**) Spearman correlation analysis between differential bacterial genera and the ketone body metabolites β-hydroxybutyrate, acetoacetate, and acetone; (**B**) Relative abundance of β-hydroxybutyrate in the mouse intestine of each group. Different lowercase letters indicate statistically significant differences, *p* < 0.05.

To investigate the specific gut microbiota involved in ketone body metabolism, Spearman correlation analysis was performed between the identified differential bacterial genera and three ketone body metabolites β-hydroxybutyrate, acetoacetate, and acetone (Figure 10A). β-Hydroxybutyrate and acetoacetate showed significant positive correlations with beneficial bacteria such as *Akkermansia* and *Lactobacillus*, and significant negative correlations with potentially pathogenic bacteria such as *Romboutsia* and genera associated with lipid metabolism disorders (*p* < 0.05). In contrast, acetone, a byproduct of ketone body metabolism, showed significant negative correlations with the above-mentioned beneficial bacteria and positive correlations with potentially pathogenic bacteria such as *Romboutsia*. Intestinal β-hydroxybutyrate abundance was significantly elevated in the NF group relative to AFLD (*p* < 0.05), whereas microbiota depletion in the NF-ABX group reduced β-hydroxybutyrate to levels comparable to AFLD (Figure 10B). These findings indicate that the combined intervention promotes β-hydroxybutyrate synthesis through enrichment of beneficial taxa such as *Akkermansia* and *Lactobacillus*, thereby potentially activating HCAR2-mediated anti-inflammatory and lipid-regulatory signaling.

## 4. Discussion

The pathological process of ALD encompasses alcoholic hepatitis, liver fibrosis, and cirrhosis, severely affecting patients’ quality of life [33]. Despite its substantial clinical burden, safe and effective interventions that target the gut–liver axis remain limited, and current management largely depends on alcohol abstinence and supportive care. The intestinal barrier, gut microbiota, and microbial metabolites are increasingly recognized as critical modulators of hepatic inflammation in ALD [34], yet whether specific nutritional compounds and probiotic strains can act cooperatively through a microbiota-dependent mechanism to attenuate alcohol-induced liver injury remains incompletely understood. In the present study, we used a chronic ethanol-fed mouse model to investigate whether combined niacin and *B. animalis* subsp. *lactis* F1-7 supplementation protects against alcohol-induced hepatic and intestinal injury, and to define the underlying mechanism. In the present work, Serum inflammatory parameters were conspicuously elevated in the AFLD group. Furthermore, NLRP3 inflammasome activation was observed in the liver and intestine, triggering caspase-1-dependent pyroptosis and downstream pro-inflammatory cytokine secretion. These events represent a core pathological step in the progression of ALD from steatosis to inflammation and fibrosis [35]. Concurrent delivery of niacin and *B. animalis* subsp. *lactis* F1-7 (NF) substantially suppressed circulating ALT and AST activities, mitigated inflammatory infiltrates and lipid droplet deposition within hepatic tissue in mice. In this study, HCAR2 expression was markedly increased, whereas NLRP3 expression was reduced in the NF group. NLRP3 inflammasome activation and caspase-1-dependent pyroptosis have been demonstrated in liver biopsies from patients with alcoholic hepatitis, and IL-1β and IL-18 levels correlate with disease severity and mortality. HCAR2 signaling is recognized to impede NLRP3 inflammasome priming and assembly by restricting NF-κB nuclear shuttling, thereby attenuating transcriptional induction of NLRP3 and pro-IL-1β [36]; our results are consistent with these mechanisms. Notably, after antibiotic depletion of the gut microbiota, the ameliorative effects of NF on hepatic and intestinal inflammation almost disappeared, underscoring an essential contribution of the intestinal microbial consortium to the observed protection.

Intact intestinal barrier function is essential for maintaining gut homeostasis and preventing endotoxin translocation [37]. Prolonged ethanol consumption suppresses the expression of tight junction components ZO-1, claudin-1, and occludin, thereby undermining intestinal permeability control and amplifying hepatic damage [38]. Increased intestinal permeability, reduced expression of ZO-1, claudin-1, and occludin, and elevated circulating LPS are well-documented in patients with alcohol-associated liver disease, and these correlate with hepatic inflammation and disease progression [39]. In the present study, the three barrier-associated proteins were substantially down-regulated in the AFLD cohort. Although niacin or *B. animalis* subsp. *lactis* F1-7 administered singly were each capable of augmenting their expression, the NF regimen achieved significantly more pronounced restoration than either agent alone, pointing to an additive effect. After antibiotic treatment, the levels of these three proteins were markedly decreased compared with the NF group, further confirming the necessity of an intact gut microbiota for intestinal barrier repair. As the first barrier in direct contact with alcohol, a healthy or specific gut microbiota is more conducive to resisting alcohol-induced damage and maintaining homeostasis [40]. Studies have reported dysbiosis in ALD patients, including reduced *Bifidobacterium* and *Lactobacillus* and altered levels of metabolites, and that these changes are associated with disease severity [41]. *Faecalibaculum*, an anaerobic genus almost depleted in the AFLD group, was markedly increased in the F1-7 and NF groups. This bacterium promotes liver repair by increasing the abundance of butyrate-producing bacteria and butyrate content, thereby inhibiting the overgrowth of opportunistic enteric pathogens induced by alcohol [42]. In addition, *Bacteroides* and *Bifidobacterium* promote short-chain fatty acid production, whereas a decrease in *Coriobacteriaceae_UCG-002* helps reduce lipid accumulation [43]. The results of our study confirmed that the combined intervention effectively improved the microbiota composition as reported in the literature. In the NF-ABX group, the levels of *Akkermansia* and *Dubosiella* were significantly upregulated, indicating that antibiotics caused marked gut dysbiosis. Through analysis of the metabolic cross-feeding relationships among gut microbes, microbial changes in the NF-ABX group, and Spearman correlations, our findings collectively demonstrate that the protective effects of niacin and F1-7 on the intestinal barrier mainly depend on an intact gut microecological environment.

Untargeted metabolomics showed that the NF group shifted the alcohol-disrupted intestinal metabolic profile, as evidenced by the clear separation of the NF group from the AFLD group in the PLS-DA model (Figure 8A) and by the 1101 differentially abundant metabolites identified between these two groups (Figure 8B). Enrichment analysis of differential metabolic pathways revealed significant alterations in ketone body metabolism, involving β-hydroxybutyrate. As an endogenous ligand of HCAR2, β-hydroxybutyrate engages HCAR2 present on colonic epithelial and immune cell populations, eliciting anti-inflammatory cytokine output, tempering NF-κB pathway activity, reinforcing epithelial barrier competence, and curtailing endotoxin leakage [13]. β-hydroxybutyrate specifically inhibits NLRP3 inflammasome assembly and activation [44].

Broad-spectrum antibiotics can independently alter hepatic bile acid metabolism, intestinal morphology, and ethanol absorption, and in some alcohol feeding models, antibiotic treatment alone reduces hepatic steatosis and inflammation [45,46]. The non-specific bacteriostatic effect of antibiotics may produce complex off-target effects, suppressing not only potentially harmful bacteria but also non-target microorganisms with beneficial metabolic functions, thereby interfering with the overall metabolic network of the gut ecosystem [47]. Therefore, the possibility that antibiotic-associated effects contributed to the phenotype observed in the NF-ABX group cannot be excluded, and the absence of an ABX-only control group is an important limitation. However, the primary purpose of the NF-ABX group in this study was to test whether the protective effects of the combined intervention depend on an intact resident microbial ecological context, rather than to precisely attribute the effects to the absence of any specific bacterial taxon. In this regard, the observation that the protective effects of the NF intervention were largely abolished in antibiotic-treated mice is consistent with, but does not definitively prove, a microbiota-dependent mechanism. It should also be noted that the present data do not allow us to distinguish between diminished microbial BHB production and other explanations such as altered host ketogenesis or caecal water content [48,49]. The targeted quantification of BHB and acetoacetate, together with assessment of hepatic ketogenic capacity, would help clarify the source of the intestinal BHB signal, although such analyses were not performed in the present study. Future studies incorporating an ABX-only control group, together with targeted BHB quantification, will be necessary to fully separate the loss of intervention effect from direct antibiotic effects superimposed on alcohol feeding and to resolve the origin of the intestinal BHB signal.

However, this study still has several limitations. For example, the results from the NF-ABX group can only demonstrate that an intact gut microbiota is a key factor for the combined intervention to exert its effects, but cannot identify the specific microbial mediators. In addition, further determination of the causal relationship underlying the β-hydroxybutyrate–HCAR2–NLRP3 mechanism is also very important. In future studies, we will further supplement validation experiments such as HCAR2-deficient mice, targeted metabolite supplementation, and fecal microbiota transplantation, so as to provide more conclusive evidence-based support for the combined intervention in improving alcohol-associated liver disease, and to enable metabolite-based microecological intervention strategies to be applied in clinical practice more safely and reliably.

## 5. Conclusions

In this study, a mouse model of alcohol-induced liver injury was established, and the effects of niacin, B. *animalis* subsp. *lactis* F1-7, and antibiotic-mediated gut microbiota depletion were evaluated. The results demonstrated that the combined administration of niacin and *B. animalis* subsp. *lactis* F1-7 effectively ameliorated alcohol-induced hepatic steatosis and liver function impairment, corrected serum lipid metabolism disorders, and restored intestinal barrier integrity, with effects greater than either intervention alone. The underlying mechanism was associated with the upregulation of HCAR2 and inhibition of NLRP3 inflammasome activation. These protective effects are largely dependent on the gut microbiota. Furthermore, intestinal BHB was enriched by the combined intervention in a microbiota-dependent manner, and this enrichment was associated with HCAR2 activation. Whether this BHB is of microbial or host origin, and whether it causally mediates the HCAR2 pathway, requires further investigation.

## Figures and Tables

**Table 1:** Primer sequences for RT-qPCR.

Gene Name	Forward	Reverse
*β-actin*	ACTGCTCTGGCTCCTAGCAC	CCACCGATCCACACAGAGTA
*NLRP3*	GAGCTGGACCTCAGTGACAATGC	ACCAATGCGAGATCCTGACAACAC
*ASC*	CAGCACAGGCAAGCACTCATT	TCATCTTGTCTTGGCTGGTGG
*Caspase-1*	CCAGAGCACAAGACTTCTGAC	TGGTGTTGAAGAGCAGAAAGC
*IL-1β*	TCGCAGCAGCACATCAACAAGAG	TGCTCATGTCCTCATCCTGGAAGG

## Data Availability

All data are available in the main text. For other raw data, please contact the corresponding author.
